# A Simple Model of Tetracycline Antibiotic Resistance in the Aquatic Environment (with Application to the Poudre River)

**DOI:** 10.3390/ijerph8020480

**Published:** 2011-02-15

**Authors:** Ferdi L. Hellweger, Xiaodan Ruan, Sarah Sanchez

**Affiliations:** Center for Urban Environmental Studies, Department of Civil & Environmental Engineering, Northeastern University, 360 Huntington Ave, Boston, MA 02115, USA; E-Mails: xiaodanruan@gmail.com (X.R.); sanchez.sa@husky.neu.edu (S.S.)

**Keywords:** antibiotic, antibiotic resistance, model, tetracycline, Poudre River

## Abstract

Antibiotic resistance is a major concern, yet it is unclear what causes the relatively high densities of resistant bacteria in the anthropogenically impacted environment. There are various possible scenarios (hypotheses): (A) Input of resistant bacteria from wastewater and agricultural sources is significant, but they do not grow in the environment; (B) Input of resistant bacteria is negligible, but the resistant bacteria (exogenous or endogenous) grow due to the selection pressure of the antibiotic; (C) Exogenous bacteria transfer the resistance to the endogenous bacteria and those grow. This paper presents a simple mechanistic model of tetracycline resistance in the aquatic environment. It includes state variables for tetracyclines, susceptible and resistant bacteria, and particulate and dissolved organic matter in the water column and sediment bed. The antibiotic partitions between freely dissolved, dissolved organic matter (DOM)-bound and solids-bound phases, and decays. Bacteria growth is limited by DOM, inhibited by the antibiotic (susceptible bacteria only) and lower due to the metabolic cost of carrying the resistance (resistant bacteria only). Resistant bacteria can transfer resistance to the susceptible bacteria (conjugation) and lose the resistance (segregation). The model is applied to the Poudre River and can reproduce the major observed (literature data) patterns of antibiotic concentration and resistance. The model suggests observed densities of resistant bacteria in the sediment bed cannot be explained by input (scenario A), but require growth (scenarios B or C).

## 1. Introduction

Antibiotics are an important weapon against bacterial diseases. However, after a new drug is introduced, bacteria generally develop resistance to it, and today there are many pathogens that are resistant to most antibiotics. The proliferation of antibiotic resistance is one of the most significant contemporary public health threats [1]. Hospitals are at the front line of this battle and a significant amount of research has focused on understanding the ecology of antibiotic resistance in this setting [2], but there is now also an emerging concern about the spread of resistance in the environment (e.g., surface waters) [3,4].

**Figure 1.**
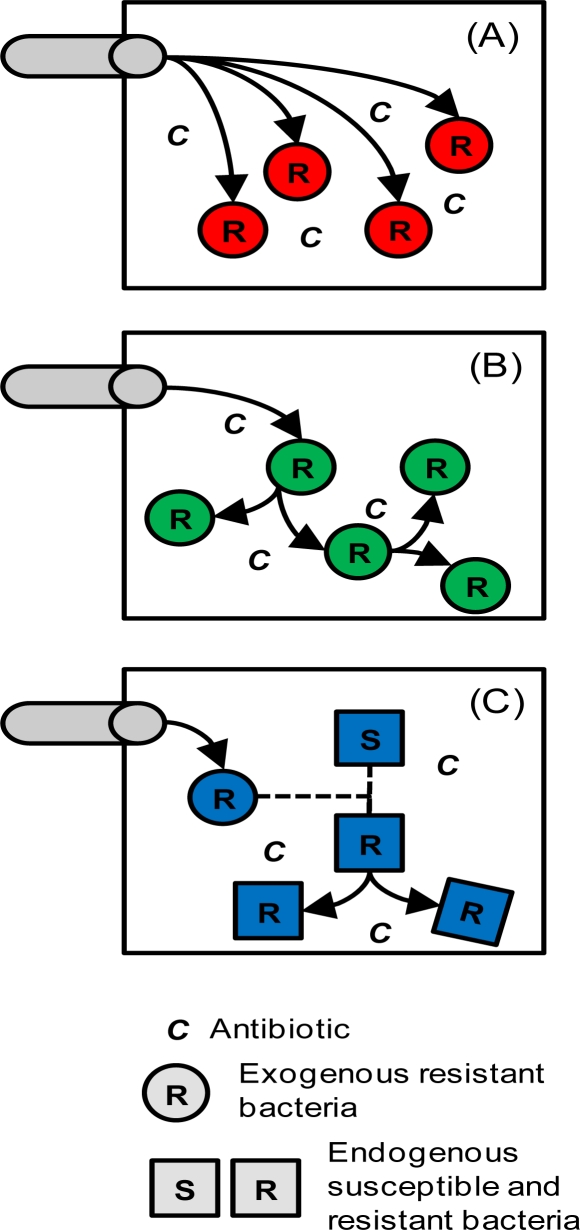
Scenarios for the presence of resistant bacteria in the environment. (A) Input of resistant bacteria from external sources is significant, but they do not grow; (B) Input of resistant bacteria is negligible, but the resistant bacteria grow due to the selection pressure of the antibiotic; (C) Exogenous bacteria transfer the resistance to the endogenous bacteria and those grow.

Tetracyclines are used extensively for human medicine and agriculture. They, and bacteria resistant to them, enter the aquatic environment from wastewater and agricultural sources, and resistance has been found to increase along rivers subject to urban and/or agricultural influences [4–7]. However, it is unclear what mechanism is responsible for the relatively high densities of resistant bacteria observed in the aquatic environment, and there are a number of possible scenarios (hypotheses, Figure 1). (A) The resistant bacteria enter from wastewater and agricultural sources, but they do not grow. Significant fractions of resistant bacteria have been found in these discharges [6,8], and enteric bacteria are typically assumed not to grow in the ambient environment. (B) The input of resistant bacteria is negligible, but the antibiotic gives them (exogenous or endogenous) a selective advantage and they grow. Here, exogenous and endogenous is meant with respect to the aquatic environment. Selection of tetracycline resistant bacteria or resistance genes under surface water conditions has been demonstrated in controlled experiments [9–11]. (C) The exogenous resistant bacteria transfer the resistance to the endogenous bacteria and those grow. Transfer of resistance plasmids (horizontal gene transfer) has been demonstrated to occur under sediment bed and water column conditions [7,12,13]. Another scenario is co-selection by other stresses (e.g., metals) [14], which is not considered here (discussed further below).

Models are important tools for understanding and managing environmental systems. Can a model, developed based on our current understanding of the various processes affecting antibiotic resistance, reproduce the observed patterns of resistant bacteria in the aquatic environment? Can it provide insight into why they are there?

Models for antibiotic fate and transport in the aquatic environment have been developed [15,16], but they have yet to be extended to include resistance. This may be because the mechanisms affecting antibiotic resistance are new to environmental modelers. Whereas many emerging contaminants can be modeled using established methods for toxic chemical fate and transport (e.g., sorption, photolysis), antibiotic resistance is subject to fundamentally new processes, including the toxic effect of the antibiotic and cost of carrying the resistance, and transfer of resistance among bacteria and loss.

This paper presents a simple model of tetracycline resistance in the aquatic environment. The model is then applied to the Poudre River and compared to literature data. A number of diagnostic simulations are performed to learn about the cause of tetracycline resistance in the river (*i.e.*, scenarios A–C outlined above).

## 2. Model Description

**Figure 2.**
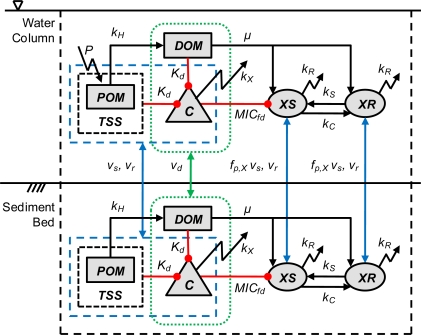
Schematic of model state variables and processes. The model includes five state variables in the water column and sediment bed: *C* = tetracycline, *XS* = susceptible bacteria, *XR* = resistant bacteria, *POM* = particulate organic matter, *DOM* = dissolved organic matter. The model includes a number of mechanisms: *k_X_* = decay, *K_d_* = partitioning, *TSS* = total suspended solids, *μ* = growth, *k_R_* = respiration, *k_C_* resistance transfer, *k_S_* = resistance loss, *MIC_fd_* = tetracycline toxicity, *P* = POM production, *k_H_* POM hydrolysis, *v_s_* = settling, *v_r_* = resuspension, *v_d_* = diffusion, *f_p,X_* = particle-associated bacteria.

The model combines concepts from existing mechanistic models of toxic chemicals, bacteria and tetracycline resistance. A schematic of model processes is presented in Figure 2. State variables include the concentration of tetracycline (*C*, μg·L^−1^), susceptible bacteria (*XS*, mgC·L^−1^), resistant bacteria (*XR*, mgC·L^−1^), particulate organic matter (*POM*, mgC·L^−1^) and dissolved organic matter (*DOM*, mgC·L^−1^) in the water column and sediment bed. There are a number of tetracyclines (tetracycline, chlortetracycline, ...) and it would be straightforward to simulate them all. However, the fate and transport properties (e.g., decay, sorption) are not known for all of them, and the model is therefore applied to the sum of tetracyclines. This is a reasonable first step that can be expanded upon in the future. Models of polychlorinated biphenyls (PCBs) have evolved in a similar manner from total to homologs to congeners. Consistent with this assumption, any literature on specific tetracyclines is assumed to apply to this model. The concentration of bacteria is defined on a biomass carbon basis (mgC·L^−1^), which is common practice in biogeochemical models [17]. This concentration can be converted to a cell density (cells·mL^−1^) assuming cell dry weight (gd·cell^−1^) and carbon fraction (gC·gd^−1^). Individual bacterial species are not resolved, although endogenous and exogenous bacteria are differentiated in one simulation. This is consistent with other water quality models [17] and reasonable considering tetracycline is a broad-spectrum antibiotic. Current models of antibiotic resistance in hospitals are at a similar level of complexity [2]. POM and DOM are simulated using single state variables, and heterogeneity in reactivity, bioavailability or sorption characteristics are not considered. In other words, all POM and DOM molecules are assumed to have the same properties. It is well-known that not all of the DOM is available for bacterial growth, and various methods for estimating this fraction are available (biodegradable dissolved organic carbon, assimilable organic carbon) [18,19]. However, for this simplistic model (steady-state, prescribed POM production), assuming one form of DOM is equivalent to simulating a constant available fraction (with appropriate adjustment in half-saturation constant, see Figure S3). Water column transport includes advection and dispersion, which is simulated using established water quality modeling approaches [20,21]. An overview of processes is presented below and the full details are presented in the Supplementary Information (SI).

### 2.1. Tetracycline

The mass balance equation (omitting transport terms for clarity, see SI for full equations) for tetracycline (*C*) is:

(1) $$\frac{\text{dC}}{\text{dt}}=-k_{X}\;C$$

where *k_X_* (day^−1^) is the decay rate constant. Equation 1 states that the tetracycline concentration changes due to decay, which removes tetracycline. Tetracycline decays by photolysis, hydrolysis and oxidation reactions [15], and some data for individual process rates are available [22]. However, most literature data only provide overall decay rate constants and solution chemistry required to apply more detailed models are not available.

Partitioning between freely dissolved, solids-bound and DOM-bound phases is simulated using linear partition coefficients [21]. For example, the freely dissolved (*i.e.*, not bound to solids or DOM) concentration (*C_fd_*, μg·L^−1^) is:

(2) $$C_{\text{fd}}=C\frac{1}{1+K_{d,\text{DOM}}\;\text{DOM}+K_{d,\text{solid}}\;\text{TSS}}$$

where *K_d,DOM_* (L·kgC^−1^) and *K_d,solid_* (L·kgS^−1^) are the DOM and solids partition coefficients, and *TSS* (mgS·L^−1^) is the total suspended solids concentration. Sorption of tetracyclines to solids and DOM involves a number of mechanisms (e.g., cation exchange and surface complexation) for which models have been developed [23]. However, for this field application, solids and DOM characteristics, and water chemistry are ill-defined, which prohibits the application of these models. Non-linear models (e.g., Freundlich Isotherm) have been used to explain tetracycline-DOM partitioning data in the literature [24]. However, all datasets reviewed here (see Table S4) exhibit linear partitioning at environmentally-relevant concentrations. Rose and Pedersen [15] also used simple solids and DOM partition coefficients for their model. Consistent with current understanding and data for partition coefficients [25], tetracycline partitions to total suspended solids (TSS), rather than the organic fraction (POM). Note that the DOM concentration is defined on a carbon-basis, which is reflected in the units of the partition coefficient. However, this should not be interpreted as hydrophobic binding being the dominant sorption mechanism (*i.e.*, *K_d,DOM_* > *K_OW_*) [25]. Tetracycline in the freely dissolved form is assumed to be bioavailable (see below). Settling (*v_s_*, m·d^−1^), resuspension (*v_r_*, m·d^−1^) and diffusion (*v_d_*, m·d^−1^) is simulated using established water quality modeling concepts [21].

### 2.2. Bacteria

The mass balance equations for susceptible bacteria (*XS*) and resistant bacteria (*XR*) are:

(3) $$\frac{\text{dXS}}{\text{dt}}=\mu_{S}\;\text{XS}-k_{R}\;\text{XS}-k_{C}\;\text{XR}\;\text{XS}+k_{S}\;\text{XR}$$

(4) $$\frac{d\text{XR}}{\text{dt}}=\mu_{R}\;\text{XR}-k_{R}\;\text{XR}+k_{C}\;\text{XR}\;\text{XS}-k_{S}\;\text{XR}$$

where *μ_S_* and *μ_R_* (day^−1^) are the specific growth rates for susceptible and resistant bacteria, *k_R_* (day^−1^) is the specific endogenous respiration rate, *k_C_* (L·mgC^−1^·day^−1^) is the transfer rate constant (gain of resistance), *k_S_* (day^−1^) is the segregation rate constant (loss of resistance). Equations 3 and 4 state that the susceptible and resistant bacteria concentrations change by growth (term 1), respiration (term 2), gain of resistance (term 3) and loss of resistance (term 4). The growth and respiration processes (terms 1 and 2) increase and decrease the bacteria concentration, respectively. The gain of resistance process (term 3) moves bacteria from the susceptible to the resistant pool. The loss of resistance process (term 4) moves bacteria from the resistant to the susceptible pool.

The growth rates for susceptible and resistant bacteria are:

(5) $$\mu_{S}=\mu_{\text{MAX}}\left(\frac{\text{DOM}}{K_{M}+\text{DOM}}\right)\left(1-\frac{C_{\text{fd}}}{{\text{MIC}}_{\text{fd}}}\right)$$

(6) $$\mu_{R}=\mu_{\text{MAX}}\left(\frac{\text{DOM}}{K_{M}+\text{DOM}}\right)(1-\alpha )$$

where *μ_MAX_* (day^−1^) is the maximum specific growth rate, *K_M_* (mgC·L^−1^) is the half-saturation constant, *C_fd_* (μg·L^−1^) is the freely dissolved tetracycline concentration, *MIC_fd_* (μg·L^−1^) is the freely dissolved minimum inhibitory concentration (MIC) (see Section 2.4), and *α* is the cost of resistance. The growth rate of susceptible and resistant bacteria accounts for the effect of nutrient concentration using a Monod saturation term. Note that DOM serves as a nutrient for the bacteria and sorption site for tetracycline. The growth rate of susceptible bacteria is reduced by the antibiotic (see next section). The growth rate of resistant bacteria is reduced to account for the metabolic cost of carrying the resistance (see next section). Gain of resistance is a second-order reaction between the susceptible and resistant bacteria, and the loss of resistance is a first-order reaction applied to the resistant bacteria (see next section). A fraction of the bacteria is associated with the suspended solids (*f_p,X_*) and settles (*f_p,X_* *v_s_*), and bacteria can be resuspended (*v_r_*).

### 2.3. Tetracycline Action, Resistance, Cost of Resistance and Transfer of Resistance

A great deal is known about the mechanisms of tetracycline action, resistance, cost of resistance and transfer of resistance [26], which are all included in a very simplified manner in this model. Tetracycline acts by inhibiting protein synthesis. It is a bacteriostatic antibiotic that exhibits a linear relationship between the specific growth rate and antibiotic concentration at subinhibitory concentrations [27], and that observation is the basis for the inhibition term in *Equation* 5. There are numerous tetracycline resistance genes that code for efflux pumps, ribosomal protection or antibiotic inactivation proteins [26]. The model does not resolve a specific mechanism, but simply assumes the growth rate of the resistant bacteria is not affected by the antibiotic (*i.e.*, *Equation* 6 does not have an inhibition term). In the absence of the antibiotic, the resistance gene does not provide an advantage to the bacteria, and can exert a metabolic cost [28]. If the gene is expressed, there is a cost associated with transcription and translation, and the resistance protein may itself be detrimental. If it is not expressed, it still has to be replicated to keep up with cell division. However, regulation and compensatory mutations can reduce or eliminate the metabolic cost of carrying the resistance [28,29]. Also, co-location of an antibiotic resistance gene with other genes (e.g., metal resistance [14]) on the same plasmid can make carrying the plasmid (with the antibiotic resistance gene) beneficial. Here, adaption and co-selection are not considered and the cost of carrying the resistance is modeled by reducing the growth rate of the resistant bacteria by a constant fraction (*α*, see *Equation* 6), which is based on an existing model [30]. Tetracycline resistance genes are most commonly associated with conjugative or mobilizable elements in plasmids or the chromosome [26]. Here, the gain and loss of resistance is based on an existing model of plasmid transfer [30]. That is, resistance is transferred when a susceptible and resistant bacteria meet (second-order reaction), and lost spontaneously (first-order reaction), as shown in Equations 3 and 4.

### 2.4. Bioavailability of Tetracycline

The model accounts for the bioavailability of the antibiotic. As discussed above, tetracyclines bind to solids and DOM, which reduces the freely dissolved, bioavailable concentration, a mechanism well-known to modify the toxicity of chemicals [31]. The antibiotic effect is therefore calculated based on the freely dissolved concentration (*C_fd_*), which is calculated from the total concentration using *Equation* 2. The parameter used to quantify the toxicity of the antibiotic is the freely dissolved MIC (*MIC_fd_*; MIC = minimum inhibitory concentration; fd = freely dissolved), rather than the total MIC, which is what is typically used to quantify antibiotic toxicity. The *MIC_fd_* is calculated from literature MIC values using the same partitioning calculation shown in *Equation* 2.

The bioavailability effect may not be significant for assessing toxicity *in vivo* (*i.e.*, the DOM is similar to that in lab growth media?). However, the DOM concentration in the aquatic environment is typically a factor of 1,000 lower than that of growth media used for MIC experiments (∼6 mgC·L^−1^ *vs.* ∼6 gC·L^−1^). Based on a simple partitioning calculation (*Equation* 2, *K_d,DOM_* in Table 1, *TSS* = 0), the freely dissolved fraction in growth media and aquatic environment is 1.0% and 91%, respectively. That means a total concentration of 1.0 mg·L^−1^ in growth media is equivalent to 11 μg·L^−1^ in the aquatic environment (the freely dissolved concentration is 10 μg·L^−1^ in both cases). The potency of tetracycline is increased 100-fold in the aquatic environment.

No data on sorption to growth media are available and the above calculation assumes the *K_d,DOM_* from sorption experiments using natural organic matter and humic acids is applicable to growth media. This is a major assumption, the effect of which is illustrated by a simulation where sorption to MIC test media is assumed negligible (*MIC_fd_* = MIC).

Several studies have explored the effect of environmental factors on the toxicity of tetracyclines. Garrett and Miller [32] did not observe a significant effect of nutrient concentration on growth rates, but concentrations were only varied by a factor of two, and other parameters (salt) were different as well. Lunestad and Goksoyr [33] found that binding to Mg and Ca ions significantly reduced toxicity. Halling-Sørensen *et al.* [34] found reduced toxicity in the presence of sludge and attributed it to partitioning. Chander *et al.* [35] found reduced toxicity in a soil-water mixture with higher tetracycline affinity. These experiments do not cover the 1,000-fold difference in DOM concentration, but are generally consistent with the partitioning mechanism.

### 2.5. Organic Matter

The mass balance equations for POM and DOM are:

(7) $$\frac{\text{dPOM}}{\text{dt}}=\frac{P}{H}-k_{H}\;\text{POM}$$

(8) $$\frac{d\text{DOM}}{\text{dt}}=k_{H}\;\text{POM}-\frac{\mu_{S}}{Y}\;\text{XS}-\frac{\mu_{R}}{Y}\;\text{XR}$$

where *P* (gC·m^−2^·day^−1^) is the areal POM production rate, *H* (m) is the water column depth, *k_H_* (day^−1^) is the POM hydrolysis rate constant, and *Y* is the yield coefficient. *Equation* 7 states that the POM concentration changes due to production (term 1) and hydrolysis (term 2). The production process (term 1) adds POM. The hydrolysis process (term 2) removes POM. *Equation* 8 states that the DOM concentration changes due to hydrolysis (term 1), and growth of susceptible and resistant bacteria (terms 2 and 3). The hydrolysis process (term 1) adds DOM. The growth process (terms 2 and 3) removes DOM. POM settles (*v_s_*) and resuspends (*v_r_*), and DOM diffuses (*v_d_*) between the water column and sediment bed.

## 3. Poudre River Application

**Figure 3.**
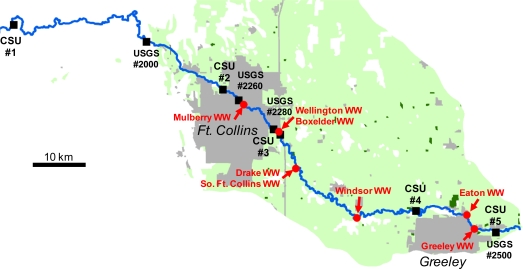
Poudre River study site showing mainstem river, sampling locations, wastewater treatment plant (WWTP) inputs and land use. CSU#1 (Colorado State University Station 1), *etc.* are sampling points of Kim and Carlson [36]. USGS (United States Geological Survey) gage numbers are abbreviated (e.g., #2260 is number 06752260). Land use colors: gray = urban, light green = agricultural (AG), dark green = confined animal feeding operations (CAFO).

The (Cache la) Poudre River in Colorado is a testbed for studying antibiotic fate and transport and antibiotic resistance in the aquatic environment (Figure 3). The watershed is a relatively complex system, including numerous discharges, withdrawals, tributaries, *etc.* Effluent from several municipal wastewater treatment plants (WWTPs) enters at various points along the river (Figure 3, red arrows). The upstream area (above USGS#2000) is mostly pristine forest. Downstream are the Ft. Collins and Greeley urban centers (gray areas), and the surrounding land use is agricultural, with mostly cropland and pasture (AG, light green) and confined animal feeding operations (CAFO, dark green).

### 3.1. Model Input

All model input and discussion is presented in the SI and only an overview is presented here. The main stem Poudre River is modeled as a steady-state, one-dimensional system with flow from WWTP, and AG and CAFO nonpoint sources. Nonpoint flow contribution from each land use type was calculated using the weighted flow accumulation function in the ArcGIS geographic information system (GIS) software. Tetracycline boundary conditions are based on site-specific data [36–40] and other literature sources. *K_d,solid_* was calibrated against the relative water column and sediment bed concentrations. The resulting value is on the low side, but within the range of literature values (Table 1), which is not surprising considering the relatively coarse-grained substrate of the river sediment bed (sand) [36]. *K_d,DOM_* was assigned as the geometric mean of the literature values (Table 1). *MIC_fd_* was assigned as the average of soil and sediment bacteria literature values (Table 1). The bacteria and organic matter parameters were assigned based on a steady-state assumption, and site-specific and literature data. Other parameters are varied among the different simulations as discussed below. A summary of calibration parameters is presented in Table S2.

### 3.2. Simulations Performed

A number of simulations with different inputs are performed. Models 1, 2 and 3 explore different inputs and decay mechanisms for tetracycline concentrations. Model 1 includes tetracycline input from wastewater treatment plants (WWTPs) only. Model 2 includes tetracycline input from WWTPs and agriculture. Model 3 includes tetracycline input from WWTPs and agricultural, and decay. Model 3 provides the best fit to the data and is adopted for three additional simulations exploring different scenarios for the presence of resistant bacteria in the river. Model 3A includes significant external input of resistant bacteria, but no growth (scenario A in Figure 1). Model 3B includes negligible input of resistant bacteria, but the resistant bacteria grow due to the selection pressure of the antibiotic (scenario B in Figure 1). Model 3C includes transfer of the resistance from the exogenous bacteria to the endogenous bacteria, and those grow (scenario C in Figure 1). Several additional diagnostic simulations are presented to investigate the role of DOM bioavailability (Model 3C1), sorption to MIC test media (Models 3B2 and 3C2) and sorption to DOM (Model 3C3), and the time-variable response after discharge is stopped (Model 3C4).

## 4. Results & Discussion

### 4.1. Tetracyclines Concentration

**Figure 4.**
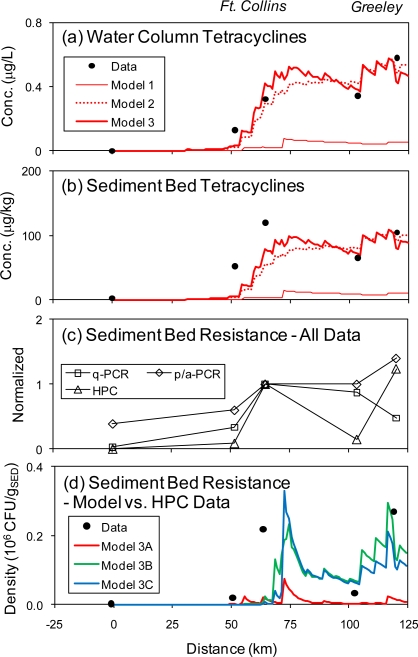
**(a)** Water column; **(b)** sediment bed tetracycline concentrations in the Poudre River; **(c–d)** Sediment bed tetracycline resistance. **Note:** In (a) and (b), Water column concentrations are dissolved. Symbols are data from Yang and Carlson [37], and Kim and Carlson [36,40]. Lines are model predictions. Model 1 includes tetracycline input from wastewater treatment plants (WWTPs) only. Model 2 includes tetracycline input from WWTPs and agriculture. Model 3 includes tetracycline input from WWTPs and agricultural, and decay. (c) Data from Pruden *et al.* [4] and Pei *et al.* [41] normalized to CSU#3 (Colorado State University Station 3) (65 km). Data are: HPC (heterotrophic plate count): mean CFUr/g_SED_ (CFU = colony forming unit) for CTC (chlortetracycline), OTC (oxytetracycline) and MCC (meclocycline), p/a-PCR (presence/absence polymerase chain reaction): resistance genes frequency of detection, q-PCR (quantitative polymerase chain reaction): (tet(W) + tet(O))/16S rRNA (ribosomal RNA) gene copies. (d) Model-data comparison. Symbols are HPC data from Pei *et al.* [41]. Lines are model predictions. All three models are based on Model 3 (tetracycline input from WWTPs and agriculture, and decay) exploring three different scenarios for the presence of resistant bacteria in the Poudre River. Model 3A includes significant external input of resistant bacteria, but no growth. Model 3B includes negligible input of resistant bacteria, but the resistant bacteria grow due to the selection pressure of the antibiotic. Model 3C includes transfer of the resistance from the exogenous bacteria to the endogenous bacteria, and those grow. Distance is downstream from CSU#1 (see Figure 3).

The water column and sediment bed concentrations of six tetracyclines, including oxytetracycline (OTC), tetracycline (TC), demeclocycline (DMC), chlortetracycline (CTC), doxycycline (DXC), meclocycline (MCC) were measured by Yang and Carlson [37], and Kim and Carlson [36,40]. The observed concentrations show an increase at Ft. Collins and another one at Greeley (Figure 4a&b). A number of model simulations are presented. Model 1 includes WWTP input based on the maximum measured tetracycline concentrations at the Ft. Collins Drake WWTP [37–39], no contribution from agricultural sources (AG, CAFO) and no decay. This model predicts an increase at Ft. Collins, but it significantly underestimates the water column and sediment bed concentrations (Figure 4a&b, thin line). This suggests significant contributions from the agricultural areas, which is reasonable considering the use of tetracyclines in agriculture. Model 2 includes WWTP inputs based on average measured tetracycline concentrations, calibrated AG and CAFO tetracycline concentrations and no decay (Figure 4a&b, dotted line). No site-specific data for runoff from agricultural areas are available, and the literature data vary greatly. The tetracycline concentration was therefore adjusted (calibrated) to match the field data. This model reproduces much of the observed spatial pattern of tetracyclines in the water column and sediment bed, including the increase in water column concentration at Ft. Collins and Greeley. However, it underestimates the increase of sediment bed concentration at Ft. Collins. The small step-like increases in concentration reflect nonpoint source inflows. Model 3 includes decay in the water column, and re-calibrated AG and CAFO concentrations (Figure 4a&b, heavy line). The decay rate constant (*k_X_*) was calibrated against the water column data and the resulting value is on the low side, but well within the range of literature values (Table 1). This is expected considering photolysis is the predominant decay mechanism, which is reduced by light attenuation in the water column [15]. The resulting calibrated concentrations for the AG and CAFO sources are within the range of the literature. The CAFO value is on the high side of the literature for agriculture, but that is expected considering the higher density of animals. Used in this manner, the model is a tool for estimating the tetracyclines concentration in runoff from agricultural lands. Model 3 provides the best fit to the data and is adopted for the simulations looking at antibiotic resistance below.

### 4.2. Tetracycline Resistance

The tretracycline resistance in the sediment bed was quantified by Pruden *et al.* [4] and Pei *et al.* [41] using three different methods, including heterotrophic plate counts (HPC; CTC, OTC and MCC), presence/absence polymerase chain reaction (p/a-PCR; *tet*B(P), *tet*(O), *tet*(S), *tet*(T) and *tet*(W)) and quantitative polymerase chain reaction (q-PCR; *tet*(O) and *tet*(W)) for various tetracycline resistance genes. The data, presented normalized to Station CSU#3 in Figure 4c, all show an increase in resistance at Ft. Collins. The HPC data decrease between Ft. Collins and Greeley and then increase again at Greeley. The q-PCR data remain relatively high and then slightly decrease at Greeley. The p/a-PCR data also remain relatively high but then increase slightly at Greeley. Note that HPC data are based on absolute densities (CFU_r_ g_SED_^−1^, CFU_r_ = colony forming unit, resistant). Normalized densities (CFU_r_/CFU_t_, CFU_t_ = colony forming unit, total) show a relatively high fraction of resistant bacteria at the most upstream station (CSU#1), which is inconsistent with the other measures of resistance (p/a-PCR and q-PCR) and may be an outlier related to the very low total plate count [41]. The low number of *tet* genes at this site was subsequently confirmed by Storteboom *et al.* [42] using p/a-PCR.

Considering the common mode of action of the tetracycline antibiotics, all of these measures are applicable to the sum of tetracyclines, but none corresponds directly to the model state variable for resistant bacteria (*XR*). The HPC data quantify tetracycline resistant bacteria, but only among the culturable bacteria. The p/a-PCR data provides an indication of the diversity of resistance genes, and it is unclear how that can be compared to model results. The q-PCR data quantify the resistant bacteria that owe their resistance to either *tet*(O) or *tet*(W), which are common resistance genes, but there are many others [26,42]. Observed resistant fractions from q-PCR data are several orders of magnitude below those from HPC data (e.g., 10^−6^ ((tet(W) + tet(O))/16S rRNA) *vs.* 10^−2^ (CFU_r_/CFU_t_) at CSU#2), suggesting that the q-PCR data do not capture all of the resistant bacteria. The model-data comparison is based on the density of culturable resistant bacteria (CFU_r_ g_SED_^−1^). This corresponds directly to the model state variable (*XR*, converted from mgC to cells as described above) if an estimate of the fraction of culturable bacteria is available. This fraction is calculated as the ratio of total culturable bacteria (CFU_t_ g_SED_^−1^) [41] to total bacteria (assumed to be 5% of sediment organic matter, see SI Section S3.3). The resulting culturable fraction is 0.058%, which is within the range of estimates for aquatic and soil environments in the literature (0.0001–1%) [43]. The model output is scaled by this factor to yield the culturable density of resistant bacteria shown in Figure 4d.

Model 3A explores the scenario that the resistant bacteria are exogenous and do not grow (scenario A). For this simulation, the fraction of resistant bacteria from WWTP, AG and CAFO sources was assigned the max. of the literature range (*f_R_* = 70%, see SI), and bacteria are entirely associated with the particles (*f_p,X_* = 100%) and do not grow (*α* = 100%). This model clearly underpredicts the sediment bed resistance, suggesting that this scenario is not plausible (Figure 4d, red line). The input of resistant bacteria is not sufficient to explain their density observed in the sediment bed.

Model 3B explores the scenario that the resistant bacteria grow in the sediment bed (scenario B). For this simulation, the resistant bacteria in the inputs is assumed to be negligible (*f_R_* = 0.1%), only reasonable fraction is associated with particles (*f_p,X_* = 10%) and they have a small cost of carrying the resistance (*α* = 1%). The cost of resistance is not constrained by the literature data and it was adjusted to match the data. The resulting value is within the range of literature values. This model can reproduce the general observed pattern of tetracycline resistance in the Poudre River (Figure 4d, green line). It underpredicts the resistance at Ft. Collins, but that can be attributed to the underprediction of tetracyclines concentration at that location. The model only includes one bacteria species and therefore does not differentiate between exogenous and endogenous bacteria. A simulation with no input of (exogenous) resistant bacteria and a trace amount of (endogenous) resistant bacteria in the sediment bed at the beginning of the simulation produces the same results.

Model 3C explores the scenario that the exogenous resistant bacteria transfer their resistance to the endogenous bacteria, which then grow (scenario C). For this model, two types of bacteria (each with susceptible and resistant forms) are simulated (see SI for details). The max growth rate of the endogenous bacteria was increased to reflect its greater fitness in the aquatic environment. Input of resistant bacteria, association with particles and cost of carrying the resistance are as in Model 3B. Transfer and segregation rate constants (*k_C_*, *k_S_*) were calibrated within the range of literature values. The model predicts transfer of the resistance to the endogenous bacteria and their growth (Figure 4d, blue line).

Model 3A cannot and Models 3B and 3C can predict the observed density of resistant bacteria in the sediment bed. What can be learned or concluded from that? The number of calibrated parameters increases with model version (see Table S2 for summary), and from a model calibration perspective the better fit of Models 3B and 3C is not surprising. However, from a mechanistic perspective, the main difference between the models is the inclusion of growth of resistant bacteria. The model without this process cannot reproduce the observations, which leads to the conclusion that this mechanism is important. It is the nature of the process (not the number of processes or parameters) that constitutes the difference between the models, upon which the conclusion is based. Simply adding another process and associated calibration parameters (e.g., predation, quorum sensing) would not make Model 3A reproduce the observations. Models 3B and 3C match the data equally well, suggesting that both scenarios are plausible. Put another way, the model does not provide insight into whether the resistant bacteria are exogenous or endogenous.

Models 3B and 3C predict selection of the antibiotic resistant bacteria at porewater concentrations of about 0.5 μg·L^−1^ (*C_d_* ≈ *C_fd_* at these DOM concentrations, corresponding to 100 μg·kgS^−1^, Figure 4b). At first glance, it appears unlikely that these relatively low concentrations, or those observed in other aquatic systems (≤1 μg·L^−1^) [44] can significantly inhibit bacterial growth, given the much higher MIC (∼1.8 mg·L^−1^) [45]. What mechanisms lead to the selection of resistant bacteria at these low concentrations? One potential reason is that environmental bacteria are significantly more susceptible than clinical pathogens, but this is not supported by the literature (Table S6). Another possibility is co-selection of antibiotic resistant bacteria by other stresses (e.g., metals) [14]. The model does not include this mechanism and therefore provides no direct evidence on the importance of this process. However, the model can reproduce the observations without this mechanism, which suggests that it may not necessarily be significant. There are two factors that lead to the selection of resistant bacteria in the model. First, as outlined above, the lower DOM in the environment increases the bioavailability by a factor of about 100. As pointed out above, this is based on the assumption that the partitioning characteristics of growth media and natural organic matter or humic acids are the same. A diagnostic simulation based on the assumption that sorption to MIC test media is negligible (*MIC_fd_* = MIC) cannot predict a significant density of resistant bacteria (Models 3B2 and 3C2, see Figure S4). Second, even at subinhibitory concentrations, the growth rate is reduced (see *Equation* 5). Unlike in laboratory, pure-culture MIC tests, bacteria in the environment are subject to competition, and small reductions in growth rate can lead to extinction by competitive exclusion (soft selection). Selection of tetracycline resistant bacteria at concentrations a factor of 10 below the MIC have been observed [46].

Several experiments have examined selection by tetracycline in the aquatic environment. In chemostats, Munoz-Aguayo *et al.* [10] observed selection at high (800 μg·L^−1^), but not low (8 μg·L^−1^) dosage, suggesting that 8 μg·L^−1^ does not exert selection pressure. However, that study included 10% growth media in the chemostat feed, which would have bound a lot of the antibiotic (90%, calculated as above). Knapp *et al.* [11] observed selection of tetracycline resistance genes in mesocosms with 20 μg·L^−1^, but not 5 μg·L^−1^. Nygaard *et al.* [9] observed selection in sediments with 50 mg·kg^−1^. The porewater concentration in these experiments is not known, but a reasonable estimate is 60 μg·L^−1^ (using log *K_d,solid_* = 2.9, geometric mean of literature values, Table S3). These experiments are consistent with selection at concentrations significantly below the MIC, but they do not demonstrate it at the porewater concentration predicted for the Poudre River. More research is needed to increase our understanding of the effect of low levels of antibiotics on bacteria, and the role of the environmental conditions.

Two additional diagnostic simulations are performed to learn about tetracycline resistance in the Poudre River. A simulation with no DOM sorption suggest that this process is not significant (Figure S5), which is reasonable considering the low DOM-bound fraction at ambient DOM concentrations (see discussion above). A simulation was performed where the input of antibiotic was stopped. This simulation is meaningful in a management context, although it is overly optimistic as it assumes immediate compliance and neglects the dynamics of tetracyclines in the watershed soils. The model predicts reduction of sediment bed resistance with a half-life of 1.4 years (Figure S6). According to the model, the river sediments should respond relatively quickly to any changes in antibiotic input.

## 5. Outlook

Understanding of tetracycline resistance in the Poudre River would benefit from additional sampling. Specifically, the AG and CAFO concentrations should be constrained with site-specific data. Characterization of the water chemistry may improve the partitioning model, or allow for application of a more mechanistically detailed model [23]. Also, the sorption characteristics of growth media used in MIC experiments (which affects the *MIC_fd_* parameter) need to be characterized. Modeling of individual tetracyclines is a natural next step. Bacteria species analysis to differentiate exogenous and endogenous strains (e.g., *Enterobacteriaceae* vs. *Aeromonas* spp.) [5] may help differentiate scenarios B and C. There is also a need to confirm the HPC estimate at the pristine location. Application of the model to another field site, with different characteristics, would help test the general applicability of the model.

There are several possible improvements or expansions of the model. The model was developed for tetracyclines, but many of the concepts are applicable or adaptable to other antibiotics. The action of the broad-spectrum bacteriostatic chloramphenicol could be modeled using the existing equations, whereas for the bactericidal kanamycin a first-order kill rate would have to be added. For chromosomally encoded resistance (e.g., *gyrA* mutation, ciprofloxacin), point mutation rather than horizontal gene transfer would need to be added. A mechanism that may be important in the environment, is co-selection by other antibiotics or metals, because the corresponding resistance genes are often located on the same plasmid [14], which could be added.

Those tasks outlined above should lead to improved understanding of antibiotic resistance in the environment. From a more basic science perspective, there is also an opportunity to use the model to explore gene ecology. So much more is known about tetracycline resistance than what is included in the model. This includes, for example, uptake and excretion (TetA mediated efflux) of tetracycline [47]. We are presently working on incorporating several of these mechanisms using the systems bioecology modeling approach [48].

## Supplementary Information



## Figures and Tables

**Table 1.** Model parameters, their units, value assigned in the model and literature range (a).

**symbol**	**units**	**value**	**literature(b)**
*K_d,solid_*	log L·kgS^−1^	2.3	−0.52–5.5
*K_d,DOM_*	log L·kgC^−1^	4.2	3.2–5.4
*k_X_*	d^−1^	0, 1.0(c)	0.046–43 (d)
*MIC_fd_*	μg·L^−1^	13	12–14
*α*	%	1.0, 100 (e)	−3.7–89
*k_S_*	d^−1^	0, 4.0 × 10^−3^(f)	0–0.13
*k_C_*	L·mgC^−1^·d^−1^	0, 1.0 × 10^−5^(f)	0–1.0

(a) Selected parameters, see SI for full list;

(b) See SI for specific references;

(c) Model 3;

(d) In water column only;

(e) Model 3A;

(f) Model 3C.

## References

[1] Levy SB, Marshall B (2004). Antibacterial resistance worldwide: Causes, challenges and responses. Nat. Med.

[2] Webb GF, D’Agata EMC, Magal P, Ruan S (2005). A model of antibiotic-resistant bacterial epidemics in hospitals. PNAS.

[3] Kümmerer K (2004). Resistance in the environment. J. Antimicrob. Chemother.

[4] Pruden A, Pei R, Storteboom H, Carlson KH (2006). Antibiotic resistance genes as emerging contaminants: Studies in Northern Colorado. Environ. Sci. Tech.

[5] Goñi-Urriza M, Capdepuy M, Arpin C, Raymond N, Caumette P, Quentin C (2000). Impact of an urban effluent on antibiotic resistance of riverine *Enterobacteriaceae* and *Aeromonas* spp. Appl. Environ. Microbiol.

[6] Tao R, Ying G-G, Su H-C, Zhou H-W, Sidhu JPS (2010). Detection of antibiotic resistance and tetracycline resistance genes in Enterobacteriaceae isolated from the Pearl rivers in South China. Environ. Poll.

[7] Grabow WOK, Prozesky OW, Burger JS (1975). Behaviour in a river and dam of coliform bacteria with transferable or non-transferable drug resistance. Water Res.

[8] Haack BJ, Andrews RE (2000). Isolation of Tn916-like conjugal elements from swine lot effluent. Can. J. Microbiol.

[9] Nygaard K, Lunestad BT, Hektoen H, Berge JA, Hormazabal V (1992). Resistance to oxytetracycline, oxolinic acid and furazolidone in bacteria from marine sediments. Aquaculture.

[10] Muñoz-Aguayo J, Lang KS, LaPara TM, González G, Singer RS (2007). Evaluating the effects of chlortetracycline on the proliferation of antibiotic-resistant bacteria in a simulated river water ecosystem. Appl. Environ. Microbiol.

[11] Knapp CW, Engemann CA, Hanson ML, Keen PL, Hall KJ, Graham DW (2008). Indirect evidence of transposon-mediated selection of antibiotic resistance genes in aquatic systems at low-level oxytetracycline exposures. Environ. Sci. Technol.

[12] Ashelford KE, Fry JC, Day MJ, Hill KE, Learner MA, Marchesi JR, Perkins CD, Weightman AJ (1997). Using microcosms to study gene transfer in aquatic habitats. FEMS Microbiol. Eco.

[13] Stewart KR, Koditschek L (1980). Drug-resistance transfer in *Escherichia coli* in New York bight sediment. Mar. Poll. Bull.

[14] Baker-Austin C, Wright MS, Stepanauskas R, McArthur JV (2006). Co-selection of antibiotic and metal resistance. Trends Microbiol.

[15] Rose PE, Pedersen JA (2005). Fate of oxytetracycline in streams receiving aquaculture discharges: Model simulations. Environ. Toxicol. Chem.

[16] Anderson PD, D’Aco VJ, Shanahan P, Chapra SC, Buzby ME, Cunningham VL, DuPlessie BM, Hayes EP, Mastrocco FJ, Parke NJ, Rader JC, Samuelian JH, Schwab BW (2004). Screening analysis of human pharmaceutical compounds in U.S. surface waters. Environ. Sci. Technol.

[17] Connolly JP, Coffin RB, Landeck RE, Hurst CJ (1992). Modeling carbon utilization by bacteria in natural water systems. Modeling the Metabolic and Physiologic Activities of Microorganisms.

[18] Servais P, Anzil A, Ventresque C (1989). A simple method for the determination of biodegradable dissolved organic carbon in water. Appl. Environ. Microbiol.

[19] Vital M, Hammes F, Egli T (2008). *Escherichia coli* O157 can grow in natural fresh water at low carbon concentrations. Environ. Microbiol.

[20] Schnoor JL (1996). Environmental Modeling: Fate and Transport of Pollutants in Water, Air and Soil.

[21] Chapra SC (1997). Surface Water-Quality Modeling.

[22] Werner JJ, Arnold WA, McNeill K (2006). Water hardness as a photochemical parameter: Tetracycline photolysis as a function of calcium concentration, magnesium concentration, and pH. Environ. Sci. Tech.

[23] Figueroa RA, Leonard A, MacKay AA (2004). Modeling tetracycline antibiotic sorption to clays. Environ. Sci. Technol.

[24] Sithole BB, Guy RD (1987). Models for tetracycline in aquatic environments. Water, Air, Soil Pollut.

[25] Tolls J (2001). Sorption of veterinary pharmaceuticals in soils: A review. Environ. Sci. Technol.

[26] Chopra I, Roberts M (2001). Tetracycline antibiotics: Mode of action, applications, molecular biology, and epidemiology of bacterial resistance. Microbiol. Mol. Biol. Rev.

[27] Garrett ER, Miller GH, Brown MRW (1966). Kinetics and mechanisms of action of antibiotics on microorganisms V chloramphenicol and tetracycline affected Escherichia coli generation rates. J. Pharm. Sci.

[28] Björkman J, Andersson DI (2000). The cost of antibiotic resistance from a bacterial perspective. Drug Resist. Updat.

[29] Bouma JE, Lenski RE (1988). Evolution of a bacterial/plasmid association. Nature.

[30] Stewart FM, Levin BR (1977). The population biology of bacterial plasmids: A priori conditions for the existence of conjugationally transmitted factors. Genetics.

[31] Di Toro DM, Zarba CS, Hansen DJ, Berry WJ, Swartz RC, Cowan CE, Pavlou SP, Allen HE, Thomas NA, Paquin PR (1991). Technical basis for establishing sediment quality criteria for nonionic organic chemicals using equilibrium partitioning. Environ. Toxicol. Chem.

[32] Garrett ER, Miller GH (1965). Kinetics and mechanisms of action of antibiotics on microorganisms III. Inhibitory action of tetracycline and chloramphenicol on *Escherichia coli* established by total and viable counts. J. Pharm. Sci.

[33] Lunestad BT, Goksoeyr J (1990). Reduction in the antibacterial effect of oxytetracycline in sea water by complex formation with magnesium and calcium. Dis. Aquat. Organ.

[34] Halling-Sørensen B, Sengeløv G, Ingerslev F, Jensen LB (2003). Reduced antimicrobial potencies of oxytetracycline, tylosin, sulfadiazin, streptomycin, ciprofloxacin, and olaquindox due to environmental processes. Arch. Environ. Cont. Toxicol.

[35] Chander Y, Kumar K, Goyal SM, Gupta SC (2005). Antibacterial activity of soil-bound antibiotics. J. Environ. Qual.

[36] Kim SC, Carlson K (2007). Temporal and spatial trends in the occurrence of human and veterinary antibiotics in aqueous and river sediment matrices. Environ. Sci. Tech.

[37] Yang S, Carlson K (2003). Evolution of antibiotic occurrence in a river through pristine, urban and agricultural landscapes. Water Res.

[38] Yang S, Carlson K (2004). Routine monitoring of antibiotics in water and wastewater with a radioimmunoassay technique. Water Res.

[39] Yang S, Cha J, Carlson K (2005). Simultaneous extraction and analysis of 11 tetracycline and sulfonamide antibiotics in influent and effluent domestic wastewater by solid-phase extraction and liquid chromatography-electrospray ionization tandem mass spectrometry. J. Chrom. A.

[40] Kim SC, Carlson K (2007). Quantification of human and veterinary antibiotics in water and sediment using SPE/LC/MS/MS. Anal. Bioanal. Chem.

[41] Pei R, Kim S-C, Carlson KH, Pruden A (2006). Effect of river landscape on the sediment concentrations of antibiotics and corresponding antibiotic resistance genes (ARG). Water Res.

[42] Storteboom H, Arabi M, Davis JG, Crimi B, Pruden A (2010). Identification of antibiotic-resistance-gene molecular signatures suitable as tracers of pristine river, urban, and agricultural sources. Environ. Sci. Technol.

[43] Handelsman J (2004). Metagenomics: Application of genomics to uncultured microorganisms. Microbiol. Mol. Biol. Rev.

[44] Kolpin DW, Furlong ET, Meyer MT, Thurman EM, Zaugg SD, Barber LB, Buxton HT (2002). Pharmaceuticals, hormones, and other organic wastewater contaminants in U.S. streams, 1999−2000: A national reconnaissance. Environ. Sci. Tech.

[45] Bryskier A (2005). Antimicrobial Agents: Antibacterials and Antifungals.

[46] Lebek G, Egger R (1983). R-selection of subbacteriostatic tetracyclin-concentrations. Zentralbl. Bakteriol. Mikrobiol. Hyg. A.

[47] Thanassi DG, Suh GS, Nikaido H (1995). Role of outer membrane barrier in efflux-mediated tetracycline resistance of *Escherichia coli*. J. Bacteriol.

[48] Hellweger FL (2010). Resonating circadian clocks enhance fitness in cyanobacteria *in silico*. Ecol. Model.

