# Supplementary material for: A Simple Model of Tetracycline Antibiotic Resistance in the Aquatic Environment (with Application to the Poudre River)
Source: Int J Environ Res Public Health. 2011 Feb 15;8(2):480–97. doi: 10.3390/ijerph8020480 (PMC3084473; doi:10.3390/ijerph8020480)
Supplement: Supplementary file 2 [file ijerph-08-00480-s002.pdf]

Supplementary Information

Sections

S1. Full model equations S2
S1.1. Water Column Advection, Dispersion and External Input . S2
S1.2. Tetracycline S2
S1.3. Bacteria S3
S1.4. Organic Matter S4
S2. Model Implementation S5
S3. Model Input S5
S3.1. Segmentation and Water Column Transport . . S6
S3.2. Tetracycline S8
S3.3. Bacteria and Organic Matter S9
S4. Additional Model Results S14
S5. Symbols, Abbreviations and Units S18
S6. Supplementary References S21

Tables

S1. Summary of Model Simulations S5
S2. Summary of Calibration Parameters S6
S3. Segmentation and Water Column Transport S7
S4. Tetracycline Parameters S8
S5. Tetracycline Boundary Concentrations S9
S6. Bacteria and Organic Matter Parameters S10
S7. Bacteria and Organic Matter Boundary Conditions . . . S12
S8. Definition of Symbols, Abbreviations and Units . . . S18

Figures

S1. Transport S7
S2. Bacteria and Organic Matter Model Results S13
S3. Model Results for 10% DOM Bioavailability . . . S14
S4. Model Results for No Sorption to MIC test media . . . S15
S5. Model Results for No DOM Sorption S16
S6. Model Results for Stop Discharge Recovery. . . . S17

S1. Full Model Equations

S1.1. Water Column Advection, Dispersion and External Input

Water column advection, dispersion and external input terms are the same for all state variables. To illustrate, the equation for tetracycline in a water column segment (i) is:

$$V_{WC,i}\frac{dC_{WC,i}}{dt}=Q_{i-1,i}\,C_{WC,i-1}-Q_{i,i+1}\,C_{WC,i}$$
$$-\frac{E_{m,i-1,i}\,A_{i-1,i}}{L_{i-1,i}}\left(C_{WC,i}-C_{WC,i-1}\right)-\frac{E_{m,i,i+1}\,A_{i,i+1}}{L_{i,i+1}}\left(C_{WC,i}-C_{WC,i+1}\right)+Q_{IN,i}\,C_{IN,i}$$

(S1)

where V_{WC} (m^3) is the water column volume (= $\Delta x\ H\ B$, see Table S3), Q ($m^3\ d^{-1}$) is the flow rate, E_m ($m^2\ d^{-1}$) is the dispersion coefficient, A (m^2) is the cross sectional area, and L (m) is the mixing length. Equation S1 states that the concentration of tetracycline in water column segment i changes with advective input from the upstream segment (term 1), advective output to the downstream segment (term 2), dispersive exchange with the upstream segment (term 3), dispersive exchange with the downstream segment (term 4) and external input (term 5). In addition to these advection, dispersion and external input processes, the concentration changes due to a number of other processes. Those depend on the state variable (e.g., tetracycline *vs.* POM) and are defined below. The advection, dispersion and external input terms, and segment indices (i) are omitted from subsequent equations for clarity.

S1.2. Tetracycline

S1.2.a. Partitioning in Water Column ($\phi = 1$)

The tetracycline state variable corresponds to the total concentration in all phases. The freely dissolved (C_{fd}), dissolved (C_d, freely dissolved + DOM-bound) and particulate (C_p, solids-bound) concentrations are:

$$C_{fd,WC}=C_{WC}\frac{1}{1+K_{d,DOM}\,DOM_{WC}+K_{d,solid}TSS_{WC}} \quad \text{(S2)}$$

$$C_{d,WC}=C_{WC}\frac{1+K_{d,DOM}\,DOM_{WC}}{1+K_{d,DOM}\,DOM_{WC}+K_{d,solid}TSS_{WC}} \quad \text{(S3)}$$

$$C_{p,WC}=C_{WC}\frac{K_{d,solid}TSS_{WC}}{1+K_{d,DOM}\,DOM_{WC}+K_{d,solid}TSS_{WC}} \quad \text{(S4)}$$

S1.2.b. Partitioning in Sediment Bed ($\phi < 1$)

The effect of porosity in the sediment bed is accounted for by including the porosity in the partitioning equation. For the freely dissolved equation, for example, this is:

$$C_{fd,SED}=C_{SED}\frac{\phi}{\phi+K_{d,DOM}\,DOM_{SED}+K_{d,solid}TSS_{SED}} \quad \text{(S5)}$$

State variables are defined on a total volume basis (e.g., C_d, µg Lt^{-1}), whereas several processes depend on the concentration on a water volume basis (e.g., C'_d, µg Lw^{-1}). The water volume and total volume based concentrations are related by the porosity (e.g., $C'_d = C_d / \phi$).

S1.2.c. Mass Balances

$$V_{WC}\frac{dC_{WC}}{dt} = -v_s\,C_{p,WC}\,As + v_r\,C_{SED}\,As - v_d\left(C_{d,WC} - C'_{d,SED}\right)As - k_{X,WC}\,C_{WC}\,V_{WC} \tag{S6}$$

$$V_{SED}\frac{dC_{SED}}{dt} = +v_s\,C_{p,WC}\,As - v_r\,C_{SED}\,As - v_d\left(C'_{d,SED} - C_{d,WC}\right)As - k_{X,SED}\,C_{SED}\,V_{SED} \tag{S7}$$

As (m^2) is the surface area. Equations S6 and S8 state that the water column and sediment bed tetracycline concentrations change due to settling (term 1), resuspension (term 2), diffusive exchange (term 3) and decay (term 4). The settling process (term 1) moves tetracycline from the water column to the sediment bed. The resuspension process (term 2) moves tetracycline from the sediment bed to the water column. The diffusion process (term 3) moves tetracycline between the water column and sediment bed depending on the concentration gradient. The decay process (term 4) removes tetracycline from the water column and sediment bed.

S1.3. Bacteria

S1.3.a. Susceptible

$$\begin{aligned} V_{WC}\frac{dXS_{WC}}{dt} &= -f_{p,X}\,v_s\,XS_{WC}\,As + v_r\,XS_{SED}\,As \\ &\quad + \mu_S\,XS_{WC}V_{WC} - k_R\,XS_{WC}V_{WC} - k_C\,XR_{WC}\,XS_{WC}\,V_{WC} + k_S\,XR_{WC}\,V_{WC} \end{aligned} \tag{S8}$$

$$\begin{aligned} V_{SED}\frac{dXS_{SED}}{dt} &= f_{p,X}\,v_s\,XS_{WC}\,As - v_r\,XS_{SED}\,As \\ &\quad + \mu_S\,XS_{SED}V_{SED} - k_R\,XS_{SED}V_{SED} - k_C\,XR_{SED}\,XS_{SED}\,V_{SED} + k_S\,XR_{SED}\,V_{SED} \end{aligned} \tag{S9}$$

S1.3.b. Resistant

$$\begin{aligned} V_{WC}\frac{dXR_{WC}}{dt} &= -f_{p,X}\,v_s\,XR_{WC}\,As + v_r\,XR_{SED}\,As \\ &\quad + \mu_R\,XR_{WC}V_{WC} - k_R\,XR_{WC}V_{WC} + k_C\,XR_{WC}\,XS_{WC}\,V_{WC} - k_S\,XR_{WC}\,V_{WC} \end{aligned} \tag{S10}$$

$$\begin{aligned} V_{SED}\frac{dXR_{SED}}{dt} &= f_{p,X}\,v_s\,XR_{WC}\,As - v_r\,XR_{SED}\,As \\ &\quad + \mu_R\,XR_{SED}V_{SED} - k_R\,XR_{SED}V_{SED} + k_C\,XR_{SED}\,XS_{SED}\,V_{SED} - k_S\,XR_{SED}\,V_{SED} \end{aligned} \tag{S11}$$

Equations S8 through S11 state that the water column and sediment bed, susceptible and resistant bacteria concentrations change due to settling (term 1), resuspension (term 2), growth (term 3), respiration (term 4), gain of resistance (term 5) and loss of resistance (term 6). The settling process (term 1) moves bacteria from the water column to the sediment bed. The resuspension process (term 2) moves bacteria from the sediment bed to the water column. The growth and respiration processes (terms 3 and 4) increase and decrease the bacteria

concentration, respectively. The gain of resistance process (term 5) moves bacteria from the susceptible to the resistant pool. The loss of resistance process (term 6) moves bacteria from the resistant to the susceptible pool.

S1.3.c. Multiple Tetracyclines

If the effect is additive, the action of multiple tetracyclines (*i.e.* TC, OTC, ...) can be simulated using a modification to Equation 5 in the main text. That is, the growth rate for the susceptible bacteria is a function of each tetracycline (*j*):

$$\mu_S = \mu_{MAX}\left(\frac{DOM}{K_M + DOM}\right)\left(1 - \sum_j \frac{C(j)_{fd}}{MIC_{fd}(j)}\right) \tag{S12}$$

This feature is not used in the application presented in the paper, because the sum of tetracyclines is simulated as one antibiotic. Equation S12 states that the growth rate increases with the DOM concentration and decreases with the concentration of each tetracycline.

S1.3.d. Multiple Bacteria Species

Multiple species of bacteria (*i.e.* endogenous and exogenous) can be simulated using the above equations. To support resistance transfer between species, resistance gain terms consider transfer from all species. For example: The resistance gain for species 1 ($XS(1) \rightarrow XR(1)$) in the sediment bed, is a function of each species (*k*) (omitting other terms from Equation S11):

$$V_{SED}\frac{dXR(1)_{SED}}{dt} = +\sum_k k_C\, XR(k)_{SED}\, XS(1)_{SED} \tag{S13}$$

This feature is used in Model 3C with two species (k = 2). Equation S13 states that the gain of resistance for species 1 is a second order process between susceptible bacteria of species 1 and the resistant bacteria of all other species (incl. species 1).

S1.4. Organic Matter

S1.4.a. POM

$$V_{WC}\frac{dPOM_{WC}}{dt} = -v_s\, POM_{WC}\, As + v_r\, POM_{SED}\, As + P\, As - k_{H,WC}\, POM_{WC} V_{WC} \tag{S14}$$

$$V_{SED}\frac{dPOM_{SED}}{dt} = v_s\, POM_{WC}\, As - v_r\, POM_{SED}\, As - k_{H,SED}\, POM_{SED} V_{SED} \tag{S15}$$

Equation S14 states that the water column POM concentration changes due to settling (term 1), resuspension (term 2), production (term 3) and hydrolysis (term 4). The settling process (term 1) removes POM from the water column. The resuspension process (term 2) adds POM to the water column. The production process (term 3) adds POM to the water column. The hydrolysis process (term 4) removes POM from the water column. Equation S15 states that the sediment bed POM concentration changes due to settling (term 1), resuspension (term 2) and hydrolysis (term 3). The settling process (term 1) adds POM to the sediment bed. The resuspension process (term 2) removes POM from the sediment bed. The hydrolysis process (term 3) removes POM from the sediment bed.

S1.4.b. DOM

$$V_{WC}\frac{dDOM_{WC}}{dt}=-v_d\left(DOM_{WC}-DOM_{SED}\right)As-\frac{\mu_S}{Y}XS_{WC}V_{WC}-\frac{\mu_R}{Y}XR_{WC}V_{WC}+k_H\,POM_{WC}V_{WC} \qquad \text{(S16)}$$

$$V_{SED}\frac{dDOM_{SED}}{dt}=-v_d\left(DOM_{SED}-DOM_{WC}\right)As-\frac{\mu_S}{Y}XS_{SED}V_{SED}-\frac{\mu_R}{Y}XR_{SED}V_{SED}+k_H\,POM_{SED}V_{SED} \text{(S17)}$$

Equations S16 and S18 state that the water column and sediment bed DOM concentrations change due to diffusion (term 1), growth of susceptible and resistant bacteria (terms 2 and 3) and hydrolysis (term 4). The diffusion process (term 1) moves DOM between the water column and sediment bed depending on the concentration gradient. The growth process (terms 2 and 3) removes DOM from the water column and sediment bed. The hydrolysis process (term 4) adds DOM to the water column and sediment bed.

S2. Model Implementation

The model, named MAQUIS, is implemented in MS Excel using the Visual Basic for Applications (VBA) programming language. Differential equations are integrated using the explicit Euler method (Chapra, 1997). The model code is available from the corresponding author on request.

S3. Model Input

Table S1. Summary of model simulations (a).

model	description	notes
0	no antibiotic or resistant bacteria	(Figure S1&2)
1	max. WWTP, no NPS, no decay	BC in Table S5, $k_X = 0$
2	ave. WWTP, calibrated NPS, no decay	BC in Table S5, $k_X = 0$
3	ave. WWTP, calibrated NPS, calibrated decay	BC in Table S5, $k_{X,WC} > 0$
3A	large input of resistant bacteria, no growth	$\alpha = 1$, f_R = max., $f_{p,X} = 1$, $k_C = 0$, $k_S = 0$
3B	small input of resistant bacteria, growth	$\alpha < 1$, f_R = tr, $f_{p,X} = 0.1$, $k_C = 0$, $k_S = 0$
3C	exogenous resistant bacteria transfer resistance to endogenous bacteria and they grow	$\alpha < 1$, f_R = tr, $f_{p,X} = 0.1$, $k_C > 0$, $k_S > 0$
3C1	10% DOM bioavailability	0.1 K_M (Figure S3)
3B2 & 3C2	no sorption to MIC test media	MIC_{fd} = MIC, $\alpha = 0$ (Figure S4)
3C3	no DOM sorption	$K_{d,DOM} = 0$ (Figure S5)
3C4	stop discharge recovery	BC in Table S5 (Figure S4)

(a) BC = boundary conditions. tr = trace.

Table S2. Summary of calibration parameters (a).

model	calibrated parameters
0	-
1	$K_{d,solid}$
2	$K_{d,solid}$, AG BC, CAFO BC
3	$K_{d,solid}$, AG BC, CAFO BC, $k_{X,WC}$
3A	$K_{d,solid}$, AG BC, CAFO BC, $k_{X,WC}$
3B	$K_{d,solid}$, AG BC, CAFO BC, $k_{X,WC}$, α
3C	$K_{d,solid}$, AG BC, CAFO BC, $k_{X,WC}$, α, k_C, k_S

(a) Parameters that were adjusted to match the observed field data.
Parameter values are inherited (*i.e.* $K_{d,solid}$ was calibrated for Model 1 and kept the same for subsequent models), except for AG BC and CAFO BC, which were re-calibrated for Model 3.

S3.1. Segmentation and Water Column Transport

The segmentation is based on the USGS National Hydrography Dataset (NHD) flowline layer, which includes length attributes. Flow rates were developed as follows (see Figure S1). Using the measured discharge and drainage area of the most downstream USGS gage, an areal flow contribution was calculated. Drainage areas for each segment were developed with GIS ArcHydo routines from a digital elevation model (DEM), and the flow contribution to each segment was calculated. This includes pristine, AG and CAFO fractions estimated using the weighted flow accumulation function. GIS data were obtained from the EPA BASINS system (EPA, 2010). This is an estimate of the unaltered flow rate (*Qnps*). Then, the WWTP discharges were added (*Qww*). Finally, a net discharge/withdrawal flow was added to balance the flow at each of the USGS gages (*Qdif*). Flow rates are based on the time period July 2001 through February 2006, corresponding to the field data. Hydraulic geometry parameters (depth-flow and width-flow relationships) were derived from data at USGS#2260 (Table S3).

Table S3. Segmentation and water column transport.

symbol	units	value	notes
Segmentation			
Δx	km	1.0	produces reasonable numerical dispersion (a)
a, *b*	-	0.32, 0.32	$H = aQ^b$; from site specific analysis (b); *b*: 0.1–0.6 (c)
e, *f*	-	11, 0.22	$B = eQ^f$; from site specific analysis (b); *f*: 0.05-0.25 (c)
H_{SED}	cm	3.0	Kim and Carlson (2007a)
ϕ	-	0.30	sand (Kim and Carlson, 2007a), 0.12-0.55 (Holtz and Kovacs, 1981)
WC Transport			
Q	$m^3 s^{-1}$	varies	see text and Figure S1
E_m	$m^2 s^{-1}$	0	all dispersion is numerical (a)

(a) Δx was set to produce reasonable numerical dispersion, E_n = 170–370 $m^2 s^{-1}$ (Fischer *et al.*, 1979; Chapra, 1997).
(b) From data at USGS#2260.
(c) Chapra, 1997, p. 248.

Figure S1. Transport. (d) Tracer simulations include 1 mg L^{-1} in respective discharge.

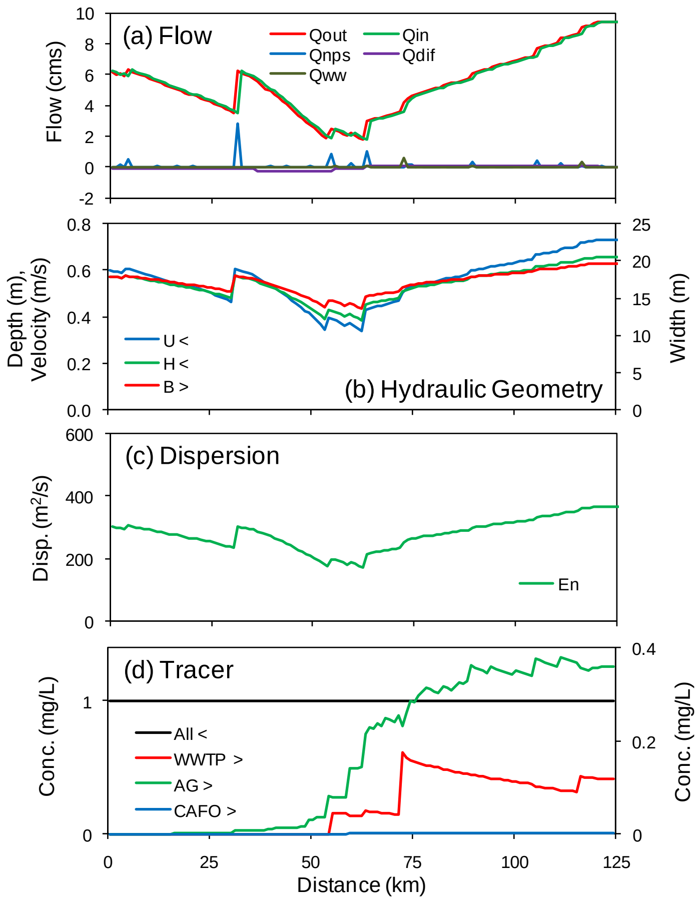

S3.2. Tetracycline

Table S4. Tetracycline parameters.

symbol	units	value	notes
$k_{X,WC}$	d^{-1}	0, 1.0(a)	oxygen/light: 0.046 (Kühne *et al.*, 2000); 0.088–0.27 (Knapp *et al.*, 2008); 0.35–38 (Verma *et al.*, 2007); 14–43 (Chen *et al.*, 2008)
$k_{X,SED}$	d^{-1}	0.0	no oxygen/light: 0 (Nygaard *et al.*, 1992); 0 (Lai *et al.*, 1995); 0 (Samuelsen *et al.*, 1994); 0.023 (Halling–Sørensen *et al.*, 2003)
$K_{d,solid}$	log L kgS^{-1}	2.3	−0.52 (b); 1.9 (c); 2.5–2.7 (d); >2.6 (e); 2.6–3.0 (f); 2.8–3.4 (g); 3.1–3.2 (h); 3.1–3.4 (i); 2.7–4.1 (j); 3.1–5.5 (k)
$K_{d,DOM}$	log L kgC^{-1}	4.2	3.2 (l); 3.6–4.2 (m); 4.5 (n); 4.4–4.7 (o); 4.8–5.4 (p)

(a) Model 3.
(b) Pouliquen and Le Bris (1996) as cited by Tolls (2001).
(c) Loke *et al.* (2002) as cited by Sarmah *et al.* (2006).
(d) Lai *et al.* (1995) as cited by Tolls (2001).
(e) Thurman and Lindsey (2000) as cited by Tolls (2001) and Sarmah *et al.* (2006).
(f) Rabolle and Spliid (2000) as cited by Tolls (2001).
(g) Smith and Samuelsen (1996) as cited by Sarmah *et al.* (2006).
(h) Sithole and Guy (1987) as cited by Tolls (2001).
(i) Gupta *et al.* (2003) as cited by Sarmah *et al.* (2006).
(j) Jones *et al.* (2005).
(k) Linear isotherms, 0.01 N $CaCl_2$, Sassman and Lee (2005).
(l) Elliot soil humic acid (ESHA), 0.01 M *I*, from data in Gu and Karthikeyan (2008).
(m) ESHA, sorption and desportion, 0.01 M NaCl, from data in Gu *et al.* (2007).
(n) Dirty Aldrich HA, MacKay and Canterbury (2005).
(o) Aldrich HA, Fit to linear portion below Ceq = 5 μM, assumed foc = 0.34, Sithole and Guy (1987).
(p) River and wetland NOM, Verma *et al.* (2007).

Table S5. Tetracycline boundary concentrations (a).

symbol	model 1	model 2	model 3 (b)	notes
WWTP	0.49	0.23	0.23	*Poudre*: ave. = 0.23, max. = 0.49 (b)<br>*Literature*:<br>< 0.050 (OTC, TC, CTC, DXC, Hirsch *et al.*, 1999);<br>0.18–0.62 (TC, Gulkowska *et al.*, 2008);<br>0.061–0.29 (TC, unfiltered, Batt *et al.*, 2007);<br>< 0.044–0.56 (TC, unfiltered, Batt *et al.*, 2006);<br>0.16–5.7 (OTC, Liu *et al.*, 2009);<br>0.70–65 (TC, Liu *et al.*, 2009)
AG	0	0.73	1.1	*Poudre*: no data<br>*Literature*:<br>0.03 (TC, Davis *et al.*, 2006);<br>0.04 (CTC, Davis *et al.*, 2006);<br>< 1.0 (OTC, De Liguoro *et al.*, 2003);<br>< 0.5–1.0 (TC+OTC, Campagnolo *et al.*, 2002);<br>< 0.5–2.0 (CTC, Campagnolo *et al.*, 2002);<br>0.45–16 (TC, Peak *et al.*, 2007);<br>< 0.35–28 (OTC, Kay *et al.*, 2004);<br>< 0.35–56 (OTC, Kay *et al.*, 2005a);<br>< 0.35–260 (OTC, Kay *et al.*, 2005b)
CAFO	0	140	220	*Poudre*: no data<br>*Literature*: see AG values above
PRIST.	0	0	0	*Poudre*: < 0.06 (Kim and Carlson, 2007a)<br>*Literature*: no data

(a) C_d, μg L^{-1}.
(b) Except Model 3C2, which is run to steady–state, then all inputs are set to zero.
(c) At Ft. Collins Drake WWTP, Yang and Carlson (2003, 2004), Yang *et al.* (2005).

S3.3. Bacteria and Organic Matter

Organic matter and bacteria parameters are established using a simulation without antibiotic or resistant bacteria (Model 0). The results are presented in Figure S2.

S3.3.a. Estimation of Sediment Bed Concentrations

Several bacteria and organic matter concentrations were derived as follows. POM_{SED} was assigned 5.0 gC Lt^{-1}, based on TSS_{SED} (see Table S6), and organic matter content (0.7%, Kim and Carlson, 2007a) and organic carbon content (40%). X_{SED} was assigned 250 mgC Lt^{-1}, based on 5% of POM_{SED} (4.7 (2.3–7.8)% of sediment OM is bacteria, Wetzel, 2001, p. 636).

Table S6. Bacteria and organic matter parameters.

symbol	units	value	notes
Bacteria			
$\mu_{MAX,WC}$	d^{-1}	2.6,<br>2.6 & 2.9 (a)	0.65−4.5 (Connolly *et al.*, 1992)
$\mu_{MAX,SED}$	d^{-1}	0.26,<br>0.26 & 0.29 (a)	see Section S3.3
K_M	mgC L^{-1}	9.1	0.20−18 (Connolly *et al.*, 1992)
Y	−	0.36	0.20−0.52 (Connolly *et al.*, 1992)
k_R	d^{-1}	0.11	0.07−0.15 (Connolly *et al.*, 1992)
MIC	mg L^{-1}	1.2(i)	marine sediment bacteria: 1.4 ±1.8 (c);<br>soil bacteria: 1.1 ±0.62 (d);<br>clinical pathogen bacteria: 1.8 ±4.2 (e)
MIC_{fd}	μg L^{-1}	13	marine sediment bacteria: 14 ±18 (c);<br>soil bacteria: 12 ±6 (d);<br>clinical pathogen bacteria: 18 ±44 (e)
α	%	1.0,<br>100 (b)	−3.7−1.2 (Bouma and Lenski, 1988, g); 0.76−4.4 (Cooper *et al.*, 1987); 4.8−5.5 (Enne *et al.*, 2005); 4.0−43 (Turner, 2004); 44 (Lenski and Bouma, 1987); 23−100 (Godwin and Slater, 1979); −74−62 (Helling *et al.*, 1981, g); 89 (Anderson, 1974)
k_S	d^{-1}	0,<br>4.0×10^{-3} (a)	0 (Lenski and Bouma, 1987); 2.5×10^{-5}−1.6×10^{-2} (Helling *et al.*, 1981); 3.9×10^{-5}−0.13 (Cooper *et al.*, 1987)
k_C	L mgC^{-1} d^{-1}	0,<br>1.0×10^{-5} (a)	0−1.0×10^{-10} (Gowland and Slater, 1984, h); 2.5×10^{-4}−2.9×10^{-2} (Turner, 2004, h); 6.8×10^{-6}−1.0×10^{0} (Levin *et al.*, 1979, h)
Solids			
TSS_{WC}	mgS Lt^{-1}	16	from USGS #2000
TSS_{SED}	kgS Lt^{-1}	1.8	$= (1 - \phi)\, \rho_S$, ρ_S = 2.6 g cm^{-3}, sand (Kim and Carlson, 2007a).
OM			
$k_{H,WC}$	d^{-1}	0.05	0.01−0.07 (HydroQual, 1998)
$k_{H,SED}$	y^{-1}	5.6	constrained by steady−state assumption (see text).
P	gC m^{-2} d^{-1}	2.3	constrained by steady−state assumption (see text).
Transp.			
v_s	m d^{-1}	6.5	constrained by steady−state assumption (see text).
$f_{p,X}$	%	10,<br>100 (b)	10 (Liu *et al.*, 2006).
v_r	mm y^{-1}	21	constrained by steady−state assumption (see text).
v_d	cm d^{-1}	0.5	0.1−1.0 (Thomann and Mueller, 1987)

(a) Model 3C.
(b) Model 3A.
(c) MIC of 4 strains, omitting resistant strain V.257, NaCl treatment (Lunestad and Goksoeyr, 1990) (f).
(d) MIC of ~20 strains, initial potency (Halling−Sørensen *et al.*, 2002, 2003) (f).
(e) MIC_{50} of 28 bacteria to various tetracyclines (Bryskier, 2005) (f).
(f) Mean ± std. dev. Converted from MIC using 6.0 gC L^{-1}.
(g) Negative value corresponds to strain with adaptive mutation.
(h) Converted using 180 fgd $cell^{-1}$, median value for *E. coli* in stationary phase, 0.5 gC $gdry^{-1}$, Loferer−Krößbacher *et al.* (1998).
(i) Not a model parameter. Provided for reference only.

S3.3.b. POM Hydrolysis and Bacteria Growth Parameters in the Sediment Bed

Assuming (1) steady-state, (2) negligible DOM diffusive flux in the sediment bed DOM balance, and (3) negligible bacteria settling and resuspension fluxes in the sediment bed bacteria balance, the POM hydrolysis flux is assimilated and then respired by bacteria:

$$k_{H,SED}\, POM_{SED} = \frac{\mu}{Y} X_{SED} = \frac{k_R}{Y} X_{SED} \qquad \text{(S18)}$$

Given site-specific values for POM_{SED} and X_{SED} and literature values for k_R and Y (see Table S6), this allows for the calculation of $k_{H,SED}$ and μ. The resulting rate constant (Table S6) is within the range for the "highly reactive component" of 1–10 y^{-1} provided by Boudreau (1997, p. 152). Bacteria growth parameters (μ_{MAX}, K_M, Y and k_R) were assigned as the midpoint of a literature summary (Connolly *et al.*, 1992) (see Table S6), with the sediment bed μ_{MAX} reduced to 10% to account for the slower growth rate in the sediment bed. That is, the DOC consumption per bacteria is typically much less in the sediment bed (e.g., Wetzel, 2001, p. 767).

S3.3.c. Settling and Resuspension Velocities

Assuming (1) steady-state, (2) no long-term net deposition/erosion of sediment bed solids, and (3) no significant solids decay, the sediment bed solids balance yields:

$$v_s\, TSS_{WC} = v_r\, TSS_{SED} \qquad \text{(S19)}$$

Assuming steady-state and combining the sediment bed POM mass balance (Equation S15) with Equation S19 yields:

$$v_s = \left(v_r + k_{H,SED} H_{SED}\right)\frac{POM_{SED}}{POM_{WC}} = \frac{k_{H,SED} H_{SED}}{\dfrac{POM_{WC}}{POM_{SED}} - \dfrac{TSS_{WC}}{TSS_{SED}}} \qquad \text{(S20)}$$

Given site-specific values for H_{SED}, TSS_{WC}, TSS_{SED}, POM_{WC}, POM_{SED} and a value for $k_{H,SED}$ (see above and Tables S1&7), this allows for calculation of v_s (Equation S4) and v_r (Equation S2). The resulting settling velocity (Table S6) is within the range of values for silt with particle diameters 10–20 μm (5.6-22 m d^{-1}, Chapra, 1997).

S3.3.d. POM Production

Assuming steady-state, the water column POM balance yields:

$$P = v_s\, POM_{WC} + k_{H,WC}\, POM_{WC} H_{WC} - v_r\, POM_{SED} \qquad \text{(S21)}$$

Given $k_{H,SED}$, v_s and v_r and site-specific values for H_{WC}, POM_{WC}, POM_{SED} (see above and Tables S1&S7), this allows for the calculation of P. The resulting autochthonous POM production is on the high side of periphyton net primary production rates summarized in the literature (0.0024–2.4, Whitton, 1975, p. 253). Increasing the POM concentration in the NPS inflow (which would be reasonable, but is not included in the simple mass balance presented in Equation S21) would reduce P and produce essentially the same model behavior (*i.e.* the model does not differentiate where the POM comes from).

Table S7. Bacteria and Organic Matter Boundary Conditions (a).

symbol	units	value	notes
WWTP			
X (b)	mgC L^{-1}	0.16	assume 5% of POM (as for sediment, see text)
f_R (b)	%	0.10, 72 (c)	0.12 (McPherson and Gealt, 1986); 1.7–3.2 (Linton *et al.*, 1974); 4 (1–9) (Grabow and Prozesky, 1973); 6.9 (Baya *et al.*, 1986); 4.0–11 (Murray *et al.*, 1984); 10 (Watkinson *et al.*, 2007b); 13 (Cooke, 1976); 21 (Watkinson *et al.*, 2007a); 25 (Mach and Grimes, 1982); 26 (Fontaine and Hoadley, 1976); 23–40 (Mezrioui and Baleux, 1994); 72 (Tao *et al.*, 2010) (d)
POM	mgC L^{-1}	3.1	based on site data (e)
DOM	mgC L^{-1}	4.6	based on site data (e)
NPS			
X (b)	mgC L^{-1}	0.020	assumed 5% of POM (as for sediment, see text); 0.008–0.45 (Wetzel, 2001, p. 493)
f_R (b)	%	0.10, 71 (c)	PRIST.: ND–0.3 (Ghosh and LaPara, 2007)<br>AG&CAFO: 0.001–0.2 (Ghosh and LaPara, 2007); 0.59–17 (Sengelov *et al.*, 2003); 26 (Sayah *et al.*, 2005); 33 (Sapkota *et al.*, 2007);<br>20–65 (Parveen *et al.*, 2006); 29–71 (Haack and Andrews, 2000) (d)
POM	mgC L^{-1}	0.40	**0.40 (0.1**–**2.1)** (USGS 06752000); **0.7**–**0.9** (USGS06752280)
DOM	mgC L^{-1}	3.2	**3.3 (1.5**–**8.4)** (USGS 06752000); **2.9**–**3.2** (USGS06752280);<br>**3.2 (1.2**–**7.7)** (USGS 404200105145600)

(a) Bold indicates Poudre River value.
(b) $X = XS + XR$. $XR = f_R \times X$.
(c) Model 3A, AG&CAFO.
(d) Literature values include any tetracycline, any bacteria. For WWTP, includes domestic wastewater from any treatment stage (e.g., influent, effluent). For AG, includes effluent, ponds and other environment.
(e) Calculated using POM = TSS × 0.4 gC gS^{-1}, DOM = TOM − POM. TOM = 8.7 mgC L^{-1}, TSS = 7.7 mg L^{-1}, average from Mulberry and Drake WWTPs for period 7/2001-2/2006.

S3.3.e. Discussion of Bacteria and Organic Matter Model Results

The bacteria and organic matter concentrations and mass balance terms are presented in Figure S2. The average water column bacteria concentration is 33 µgC L^{-1}. Warbington *et al.* (1987) measured heterotrophic bacteria concentration of 9.0 × 10^4 CFU mL^{-1} (geometric mean) in the Poudre River, which corresponds to 8.1 µgC L^{-1} (using 180 fgd $cell^{-1}$, median value for *E. coli* in stationary phase, 0.5 gC $gdry^{-1}$, Loferer-Krößbacher *et al.*, 1998). The model value is expectedly higher, since only a fraction of bacteria are culturable. The average sediment bed bacteria concentration is 260 mgC Lt^{-1}. This is slightly higher than the value used in the derivation of the organic matter parameters (see above), which reflects input from the WWTPs. Pei *et al.* (2006) observed heterotrophic plate counts (HPC) in the Poudre River of 7.8 × 10^5 CFU_t g_{SED}^{-1} (geometric mean), which corresponds to 0.15 mgC Lt^{-1} (using conversions above, ϕ and ρ_S in Table S3&6). Again, the discrepancy is consistent with only a fraction of bacteria being culturable (see also discussion in main text). The average POM

concentrations in the water column and sediment bed are 0.42 mgC L^{-1} and 5.3 gC Lt^{-1} (0.29%). These values are slightly higher than those used in the derivation of the organic matter parameters (see above), which reflects input from the WWTPs. The average DOM concentrations in the water column and sediment bed are 3.3 and 6.6 mgC Lw^{-1}, respectively. The sediment bed DOM concentration is in good agreement with observed porewater concentrations reported in the literature (5.4–21 mgC Lw^{-1}, Lin *et al.*, 2003; 3.8–24 mgC Lw^{-1}, Achman *et al.*, 1996). The assumption of negligible DOM diffusion flux in the sediment bed DOM balance and negligible bacteria settling and resuspension fluxes in the sediment bed bacteria balance are confirmed by examining the mass balance terms in Figure S2.

Figure S2. Bacteria and organic matter model results. (A) Concentrations in mgC Lt^{-1}. (B) Mass balance terms in gC m^{-2} d^{-1}. Input is positive. Advection, dispersion and external input (see Equation SX) not shown. Mass balance terms are abbreviated with corresponding kinetic variable (e.g., “kRS” is endogenous respiration of susceptible bacteria). Results are for simulation without antibiotics or resistant bacteria (Model 0).

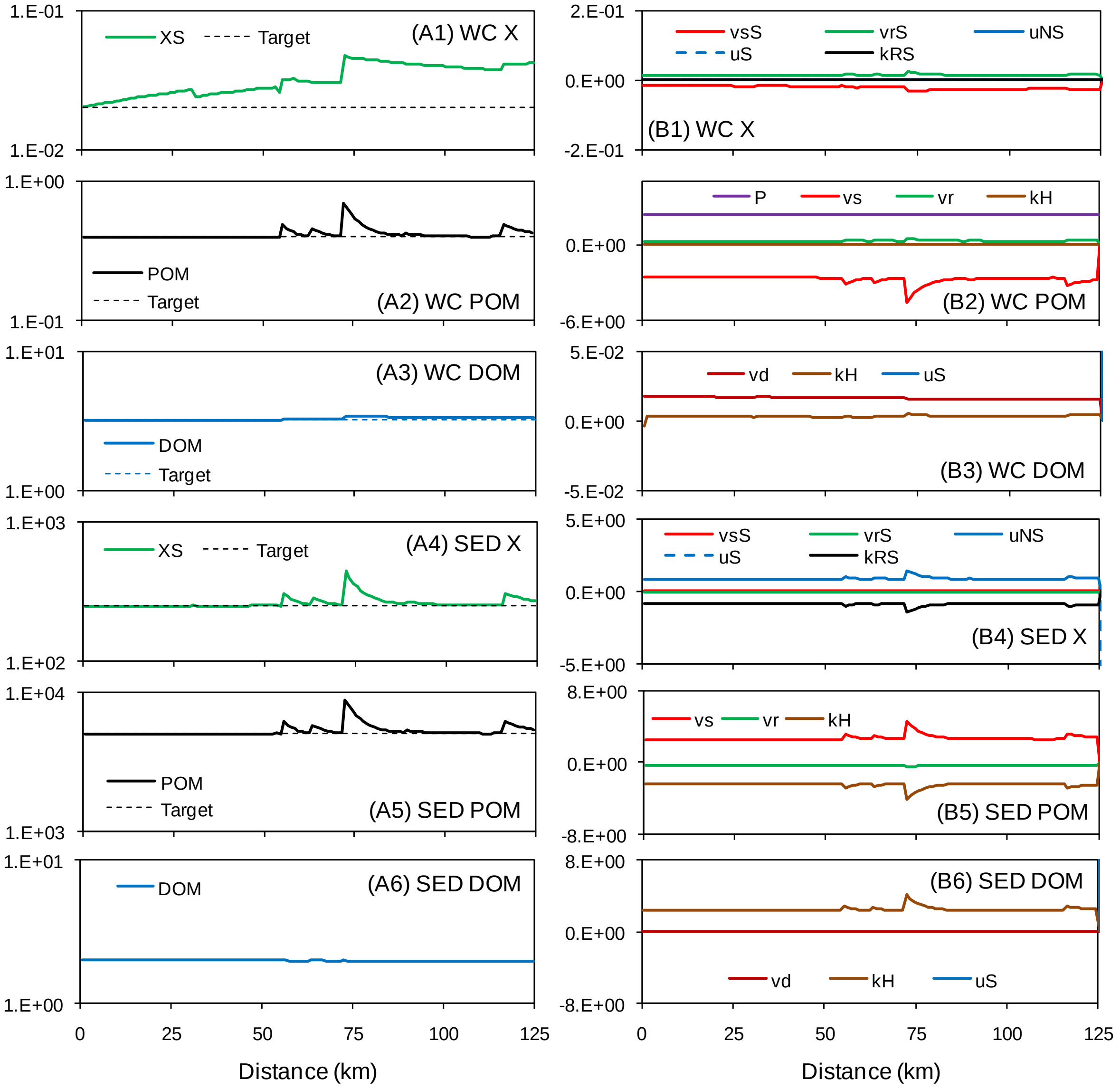

S4. Additional Model Results

Figure S3. Model results for 10% DOM bioavailability (Model 3C1). K_M parameter was adjusted to 0.91 mgC L^{-1}. Spatial pattern of (a) water column and (b) sediment bed tetracycline concentrations, and (c) tetracycline resistance in the Poudre River. Symbols are data from Yang and Carlson (2003), Kim and Carlson (2007a,b) and Pei *et al.* (2006). Lines are model predictions (see text for description of different models). Distance is downstream from CSU#1 (see Figure 3).

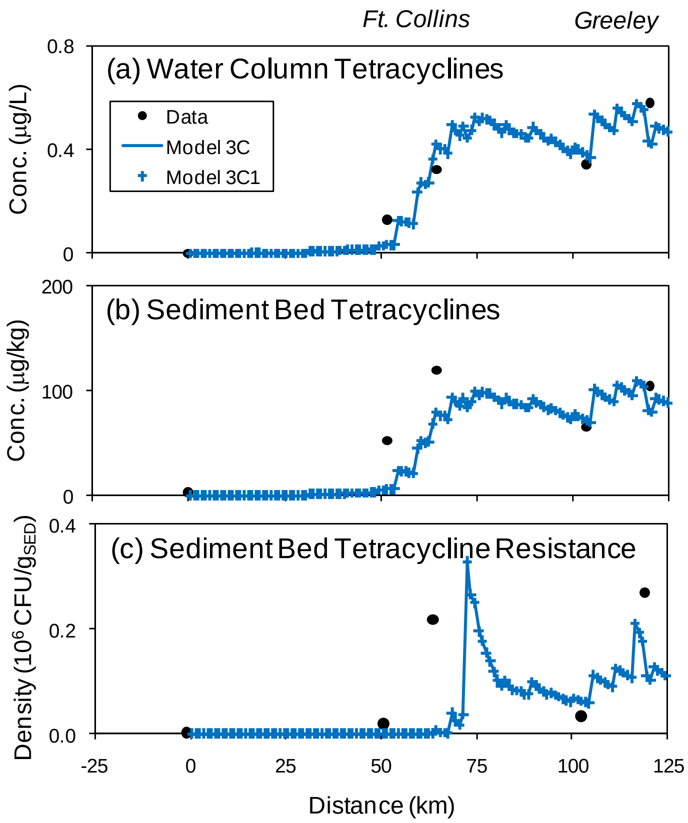

Figure S4. Model results for no sorption to MIC test media (Models 3B2 and 3C2). Spatial pattern of (a) water column and (b) sediment bed tetracycline concentrations, and (c) tetracycline resistance in the Poudre River. Symbols are data from Yang and Carlson (2003), Kim and Carlson (2007a,b) and Pei *et al.* (2006). Lines are model predictions (see text for description of different models). Distance is downstream from CSU#1 (see Figure 3).

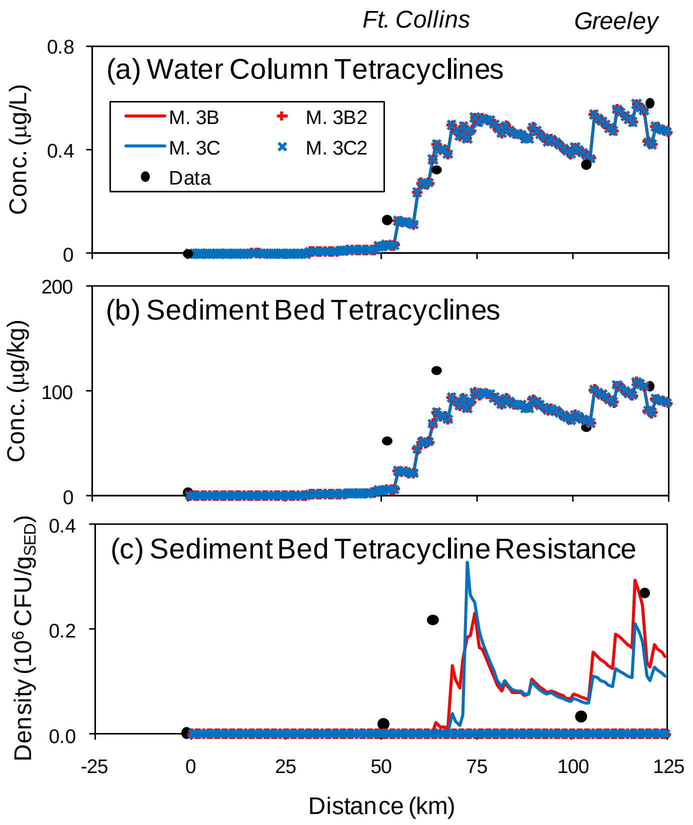

Figure S5. Model results for no DOM sorption (Model 3C3). Spatial pattern of (a) water column and (b) sediment bed tetracycline concentrations, and (c) tetracycline resistance in the Poudre River. Symbols are data from Yang and Carlson (2003), Kim and Carlson (2007a,b) and Pei *et al.* (2006). Lines are model predictions (see text for description of different models). Distance is downstream from CSU#1 (see Figure 3).

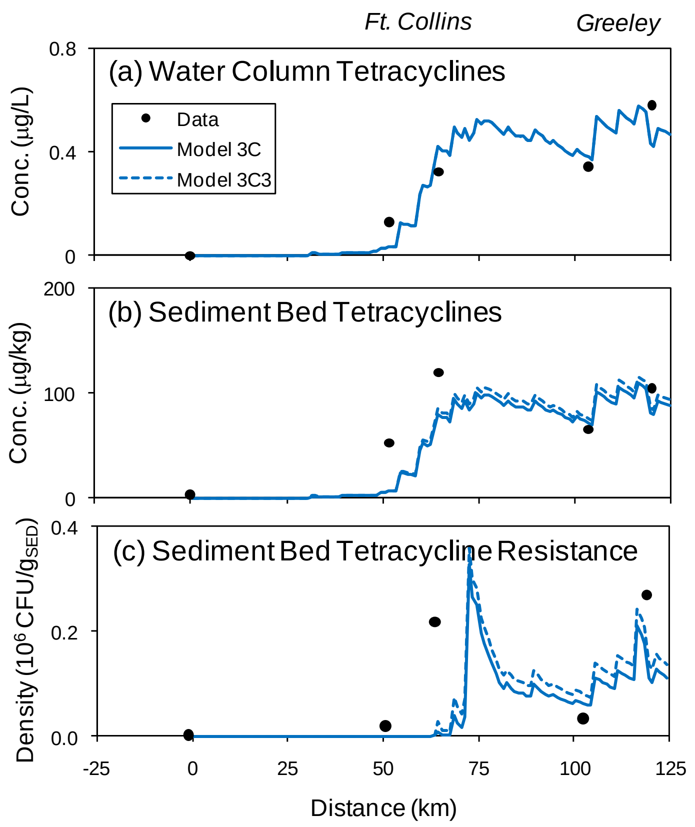

Figure S6. Model results for stop discharge recovery (Model 3C4). Temporal pattern of (a) water column and (b) sediment bed tetracycline concentrations, and (c) tetracycline resistance (note change of scale from other plots) in the Poudre River. Location is downstream of Ft. Collins (80 km, see Figure 3). Lines are model predictions (see text for description of different models). Model output interval is 0.5 years.

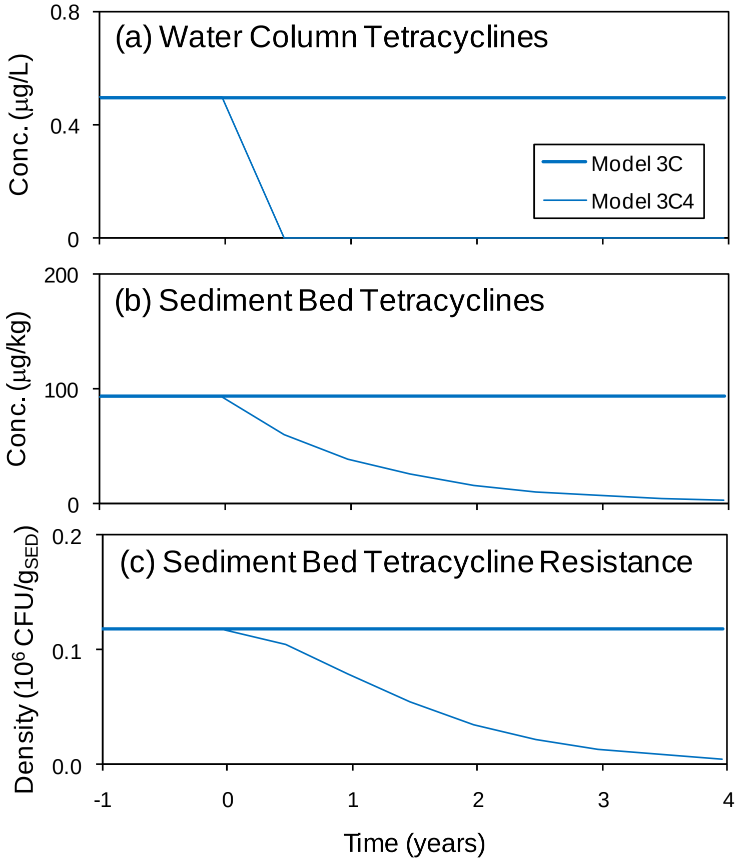

S5. Symbols, Abbreviations and Units

Table S8. Definition of symbols, abbreviations and units.

**symbol/ abbreviation/ unit**	**definition**
A	cross sectional area
a	hydraulic geometry parameter
AG	agricultural
α	cost of resistance
As	surface area
b	hydraulic geometry parameter
B	width
BASINS	Better Assessment Science Integrating point & Non-point Sources
BC	boundary condition
C	concentration of tetracycline
CAFO	confined animal feeding operations
C_d	dissolved *C* (freely dissolved + DOM-bound)
C'_d	C_d, water volume basis
Ceq	equilibrium concentration
C_{fd}	freely dissolved *C*
CFU	colony forming unit
$\mathrm{CFU_r}$	CFU, resistant
$\mathrm{CFU_t}$	CFU, total
C_p	particulate *C*
CSU	Colorado State University
CTC	chlortetracycline
Δx	segment length
DEM	digital elevation model
DMC	demeclocycline
DOC	dissolved organic carbon
DOM	dissolved organic matter
DXC	doxycycline
e	hydraulic geometry parameter
E. coli	*Escherichia coli*
E_m	dispersion coefficient, model-assigned

Table S8. *Cont.*

symbol/ abbreviation/ unit	definition
E_n	dispersion coefficient, numerical
EPA	Environmental Protection Agency
ESHA	Elliot soil humic acid
f	hydraulic geometry parameter
fd	freely dissolved
foc	fraction organic carbon
$f_{p,X}$	fraction particle-associated bacteria
f_R	fraction of resistant bacteria
g	gram
gC	g carbon
gd	g dry
GIS	geographic information system
gS	g solids
g_{SED}	g sediment
H	depth
HA	humic acid
HPC	heterotrophic plate count
i	segment index
I	ionic strength
j	tetracycline index
k	bacteria species index
k_C	transfer rate constant
K_d	partition coefficient
$K_{d,DOM}$	DOM partition coefficient
$K_{d,solid}$	solids partition coefficient
k_H	POM hydrolysis rate constant
$k_{H,SED}$	k_H, sediment bed
K_M	half-saturation constant
K_{OW}	octanol-water partition coefficient
k_R	specific endogenous respiration rate
kRS	endogenous respiration of susceptible bacteria

Table S8. *Cont.*

symbol/ abbreviation/ unit	definition
k_S	segregation rate constant
k_X	decay rate constant
$k_{X,WC}$	k_X, water column
L	mixing length
L	liter
Lt	L, total
Lw	L, water
MCC	meclocycline
MIC	minimum inhibitory concentration
MIC_{fd}	freely dissolved MIC
μ	growth rate
μ_{MAX}	maximum specific growth rate
μ_R	μ, resistant bacteria
μ_S	μ, susceptible bacteria
ND	non-detect
NHD	National Hydrography Dataset
NOM	natural organic matter
NPS	nonpoint source
OM	organic matter
OTC	oxytetracycline
P	POM production rate
p/a-PCR	presence/absence PCR
PCB	polychlorinated biphenyl
PCR	polymerase chain reaction
ϕ	porosity
POM	particulate organic matter
PRIST	pristine
Q	flow rate
Qdif	net discharge/withdrawal flow
Q_{IN}	external inflow rate

Table S8. *Cont.*

symbol/ abbreviation/ unit	definition
Qnps	unaltered flow rate
q-PCR	quantitative PCR
Qww	WWTP discharges
ρ_S	solids density
rRNA	ribosomal RNA
SED	sediment bed
SI	Supplementary Information
TC	tetracycline
TOM	total organic matter
tr	trace
TSS	total suspended solids
USGS	U.S. Geological Survey
VBA	Visual Basic for Applications
v_d	diffusion velocity
v_r	resuspension velocity
v_s	settling velocity
V	volume
WC	water column
WWTP	wastewater treatment plant
X	bacteria
XR	resistant bacteria
XS	susceptible bacteria
Y	yield coefficient

S6. Supplementary References

Achman, D.R.; Brownawell, B.J.; Zhang, L. Exchange of polychlorinated biphenyls between sediment and water in the hudson river estuary. *Estuaries* **1996**, *19*, 950-965.

Anderson, J.D. The effect of r-factor carriage on the survival of *Escherichia Coli* in the human intestine. *J. Med. Microbiol.* **1974**, *7*, 85-90.

Anderson, P.D.; D'Aco, V.J.; Shanahan, P.; Chapra, S.C.; Buzby, M.E.; Cunningham, V.L.; DuPlessie, B.M.; Hayes, E.P.; Mastrocco, F.J.; Parke, N.J.; Rader, J.C.; Samuelian, J.H.; Schwab, B.W. Screening analysis of human pharmaceutical compounds in U.S. surface waters. *Environ. Sci. Technol.* **2004**, *38*, 838-849.

Baker-Austin, C.; Wright, M.S.; Stepanauskas, R.; McArthur J.V. Co-selection of antibiotic and metal resistance. *Trends Microbiol.* **2006**, *14*, 176-182.

Batt, A.L.; Bruce, I.B.; Aga, D.S. Evaluating the vulnerability of surface waters to antibiotic contamination from varying wastewater treatment plant discharges. *Environmental Pollution* **2006**, *142*, 295-302.

Batt, A.L.; Kim, S.; Aga, D.S. Comparison of the occurrence of antibiotics in four full-scale wastewater treatment plants with varying designs and operations. *Chemosphere* **2007**, *68*, 428-435.

Baya, A.M.; Brayton, P.R.; Brown, V.L.; Grimes, D.J.; Russek-Cohen, E.; Colwell, R.R. Coincident plasmids and antimicrobial resistance in marine bacteria isolated from polluted and unpolluted Atlantic Ocean samples. *Appl. Environ. Microbiol.* **1986**, *51*, 1285-1292.

Boudreau, B.P. *Diagenetic Models and Their Implementation. Modeling Transport and Reactions in Aquatic Sediments*; Springer: Heidelberg, 1997.

Bouma, J.E.; Lenski, R.E. Evolution of a bacterial/plasmid association. *Nature* **1988**, *335*, 351-352.

Bryskier, A., Ed. *Antimicrobial Agents: Antibacterials and Antifungals*; ASM Press: Washington, USA, 2005.

Campagnolo, E.R.; Johnson, K.R.; Karpati, A.; Rubin, C.S.; Kolpin, D.W.; Meyer, M.T.; Esteban, J.E.; Currier, R.W.; Smith, K.; Thu, K.M.; McGeehin, M. Antimicrobial residues in animal waste and water resources proximal to large-scale swine and poultry feeding operations. *The Science of The Total Environment* **2002**, *299*, 89-95.

Chander, Y.; Kumar, K.; Goyal, S.M.; Gupta, S.C. Antibacterial Activity of Soil-Bound Antibiotics. *J. Environ. Qual.* **2005**, *34*, 1952-1957.

Chapra, S.C. *Surface Water-Quality Modeling*; McGraw-Hill: Boston, 1997.

Chen, Y.; Hu, C.; Qu, J.; Yang, M. Photodegradation of tetracycline and formation of reactive oxygen species in aqueous tetracycline solution under simulated sunlight irradiation, *Journal of Photochemistry and Photobiology A: Chemistry* **2008**, *197*, 81-87.

Connolly, J.P.; Coffin, R.B.; Landeck, R.E. Modeling carbon utilization by bacteria in natural water systems. In: Hurst, C. J. (ed.). *Modeling the metabolic and physiologic activities of microorganisms*. John Wiley & Sons. Inc., New York, p. 249-276, 1992.

Cooke, M.D. Antibiotic resistance in coliform and faecal coliform bacteria from natural waters and effluents. *N.Z. J. Mar. Freshwat. Res.* **1976**, *10*, 391-397.

Cooper, N.S.; Brown, M.E.; Caulcott, C.A. A Mathematical Method for Analysing Plasmid Stability in Micro-organisms. *Journal of General Microbiology* **1987**, *133*, 1871-1880.

Davis, J.G.; Truman, C.C.; Kim, S.C.; Ascough, J.C., II; Carlson, K. Antibiotic Transport via Runoff and Soil Loss. *J. Environ. Qual.* **2006**, *35*, 2250-2260.

De Liguoro, M.; Cibin, V.; Capolongo, F.; Halling-Sorensen, B.; Montesissa, C. Use of oxytetracycline and tylosin in intensive calf farming: evaluation of transfer to manure and soil. *Chemosphere* **2003**, *52*, 203-212.

Di Toro, D.M.; Zarba, C.S.; Hansen, D.J.; Berry, W.J.; Swartz, R.C.; Cowan, C.E.; Pavlou, S.P.; Allen, H.E.; Thomas, N.A.; Paquin, P.R. Technical basis for establishing sediment quality criteria for nonionic organic chemicals using equilibrium partitioning. *Environ. Toxicol. Chem.* **1991**, *10,* 1541-1583.

Enne, V.I.; Delsol, A.A.; Davis, G.R.; Hayward, S.L.; Roe, J.M.; Bennett, P.M. Assessment of the fitness impacts on Escherichia coli of acquisition of antibiotic resistance genes encoded by different types of genetic element. *Journal of Antimicrobial Chemotherapy* **2005**, *56,* 544-551.

EPA. BASINS (Better Assessment Science Integrating point & Non-point Sources). Office of Water, U.S. Environmental Protection Agency (EPA). Internet resource: http://water.epa.gov/scitech/datait/models/basins/ (accessed in 2010), 2010.

Figueroa, R.A.; Leonard, A.; MacKay, A.A. Modeling Tetracycline Antibiotic Sorption to Clays. *Environ. Sci. Technol.* **2004**, *38,* 476–483.

Fischer, H.B.; List, E.J.; Imberger, J.; Koh, R.C.Y.; Brooks, N.H. Mixing in inland and coastal waters; Academic Press: New York, 1979.

Fontaine, T.D., 3rd; Hoadley, A.W. Transferable drug resistance associated with coliforms isolated from hospital and domestic sewage. *Health Lab. Sci.* **1976**, *13,* 238-245.

Garrett, E.R.; Miller, G.H. Kinetics and mechanisms of action of antibiotics on microorganisms III. Inhibitory action of tetracycline and chloramphenicol on Escherichia coli established by total and viable counts. *Journal of Pharmaceutical Sciences* **1965**, *54,* 427-431.

Garrett, E.R.; Miller, G.H.; Brown, M.R.W. Kinetics and mechanisms of action of antibiotics on microorganisms V chloramphenicol and tetracycline affected Escherichia coli generation rates. *Journal of Pharmaceutical Sciences* **1966**, *55,* 593-600.

Ghosh, S.; LaPara, T.M. The effects of subtherapeutic antibiotic use in farm animals on the proliferation and persistence of antibiotic resistance among soil bacteria. *The ISME Journal* **2007**, *1,* 191–203.

Godwin, D.; Slater, J.H. The Influence of the Growth Environment on the Stability of a Drug Resistance Plasmid in *Escherichia coli* K12. *Journal of General Microbiology* **1979**, *111*, 201-210.

Goñi-Urriza, M.; Capdepuy, M.; Arpin, C.; Raymond, N.; Caumette, P.; Quentin, C. Impact of an Urban Effluent on Antibiotic Resistance of Riverine Enterobacteriaceae and Aeromonas spp. *Applied and Environmental Microbiology* **2000**, *66,* 125-132.

Gowland, P.C.; Slater, J.H. Transfer and stability of drug resistance plasmids in *Escherichia coli* K12. *Microbial Ecology* **1984**, *10,* 1-13.

Grabow, W.O.K.; Prozesky, O.W. Drug Resistance of Coliform Bacteria in Hospital and City Sewage. *Antimicrob. Agents Chemother.* **1973**, *3,* 175-180.

Grabow, W.O.K.; Prozesky, O.W.; Burger, J.S. Behaviour in a river and dam of coliform bacteria with transferable or non-transferable drug resistance. *Water Research* **1975**, *9*, 777-782.

Gu, C.; Karthikeyan, K.G. Sorption of the Antibiotic Tetracycline to Humic-Mineral Complexes. *J. Environ. Qual.* **2008**, *37*, 704-711.

Gu, C.; Karthikeyan, K.G.; Sibley, S.D.; Pedersen, J.A. Complexation of the antibiotic tetracycline with humic acid. *Chemosphere* **2007**, *66*, 1494-1501.

Gulkowska, A.; Leung, H.W.; So, M.K.; Taniyasu, S.; Yamashita, N.; Yeung, L.W.Y.; Richardson, B.J.; Lei, A.P.; Giesy, J.P.; Lam, P.K.S. Removal of antibiotics from wastewater by sewage treatment facilities in Hong Kong and Shenzhen, China. *Water Research* **2008**, *42*, 395-403.

Gupta, S.; Singh, A.; Kumar, K.; Thompson, A.; Thoma, D. Antibiotic losses in runoff and drainage from manure applied fields. USGS-WRRI 104G National Grants Competition, 2003.

Haack, B.J.; Andrews, R.E., Jr. Isolation of Tn916-like conjugal elements from swine lot effluent. *Can. J. Microbiol.* **2000**, *46*, 542–549.

Halling-Sørensen, B.; Sengeløv, G.; Ingerslev, F.; Jensen, L.B. Reduced Antimicrobial Potencies of Oxytetracycline, Tylosin, Sulfadiazin, Streptomycin, Ciprofloxacin, and Olaquindox Due to Environmental Processes. *Archives of Environmental Contamination and Toxicology* **2003**, *44*, 7-16.

Halling-Sørensen, B.; Sengeløv, G.; Tjørnelund, J. Toxicity of Tetracyclines and Tetracycline Degradation Products to Environmentally Relevant Bacteria, Including Selected Tetracycline-Resistant Bacteria. *Archives of Environmental Contamination and Toxicology* **2002**, *42*, 263-271.

Handelsman, J. Metagenomics: Application of Genomics to Uncultured Microorganisms. *Microbiology and Molecular Biology Reviews* **2004**, *68*, 669-685.

Helling, R.B.; Kinney, T.; Adams, J. The Maintenance of Plasmid-containing Organisms in Populations of Escherichia coli. *Journal of General Microbiology* **1981**, *123*, 129-141.

Hellweger, F.L. Resonating circadian clocks enhance fitness in cyanobacteria *in silico*. *Ecological Modelling* **2010**, *221*, 1620–1629.

Hirsch, R.; Ternes, T.; Haberer, K.; Kratz, K.-L. Occurrence of antibiotics in the aquatic environment. *The Science of The Total Environment* **1999**, *225*, 109-118.

Holtz, R.D.; Kovacs, W.D. *An Introduction to Geotechnical Engineering*; Prentice-Hall: Englewood Cliffs, NJ, 1981.

HydroQual. *A Water Quality Model for Jamaica Bay: Calibration of the Jamaica Bay Eutrophication Model (JEM)*. HydroQual: Mahwah, NJ, 1998.

Jones, A.D.; Bruland, G.L.; Agrawal, S.G.; Vasudevan, D. Factors influencing the sorption of oxytetracycline to soils. *Environmental Toxicology and Chemistry* **2005**, *24*, 761-770.

Kay. P.; Blackwell, P.A.; Boxall, A.B.A. Fate of veterinary antibiotics in a macroporous tile drained clay soil. *Environmental Toxicology and Chemistry* **2004**, *23*, 1136-1144.

Kay. P.; Blackwell, P.A.; Boxall, A.B.A. Transport of veterinary antibiotics in overland flow following the application of slurry to arable land. *Chemosphere* **2005a**, *59*, 951-959.

Kay. P.; Blackwell, P.A.; Boxall, A.B.A. Column studies to investigate the fate of veterinary antibiotics in clay soils following slurry application to agricultural land. *Chemosphere* **2005b**, *60*, 497-507.

Kim, S.C.; Carlson, K. Temporal and Spatial Trends in the Occurrence of Human and Veterinary Antibiotics in Aqueous and River Sediment Matrices. *Environmental Science & Technology* **2007a**, *41*, 50-57.

Kim, S.C.; Carlson, K. Quantification of human and veterinary antibiotics in water and sediment using SPE/LC/MS/MS. *Analytical and Bioanalytical Chemistry* **2007b**, *387*, 1301-1315.

Knapp, C.W.; Engemann, C.A.; Hanson, M.L.; Keen, P.L.; Hall, K.J.; Graham, D.W. Indirect evidence of transposon-mediated selection of antibiotic resistance genes in aquatic systems at low-level oxytetracycline exposures. *Environ. Sci. Technol.* **2008**, *42*, 5348-5353.

Kolpin, D.W.; Furlong, E.T.; Meyer, M.T.; Thurman, E.M.; Zaugg, S.D.; Barber, L.B.; Buxton, H.T. Pharmaceuticals, Hormones, and Other Organic Wastewater Contaminants in U.S. Streams, 1999−2000: A National Reconnaissance. *Environmental Science & Technology* **2002**, *36*, 1202-1211.

Kühne, M.; Ihnen, D.; Möller, G.; Agthe, O. Stability of Tetracycline in Water and Liquid Manure. *Journal of Veterinary Medicine, Series A* **2000**, *47*, 379-384.

Kümmerer, K. Resistance in the environment. *Journal of Antimicrobial Chemotherapy* **2004**, *54,* 311-320.

Lai, H.-T.; Liu, S.-M.; Chien, Y.-H. Transformation of chloramphenicol and oxytetracycline in aquaculture pond sediments. *Journal of Environmental Science and Health A* **1995**, *30,* 1897-1923.

Lebek G.; Egger R. R-selection of subbacteriostatic tetracyclin-concentrations. *Zentralbl. Bakteriol. Mikrobiol. Hyg. A* **1983**, *255,* 340-345.

Lenski, R.E.; Bouma, J.E. Effects of segregation and selection on instability of plasmid pACYC184 in Escherichia coli B. *J. Bacteriol.* **1987**, *169,* 5314-5316.

Levin, B.R.; Stewart, F.M.; Rice, V.A. The kinetics of conjugative plasmid transmission: Fit of a simple mass action model. *Plasmid* **1979**, *2,* 247-260.

Levy, S.B.; Marshall, B. Antibacterial resistance worldwide: causes, challenges and responses. *Nature Medicine* **2004**, *10,* S122 - S129.

Lin, C.-H.M.; Pedersen, J.A.; Suffet, I.H. Influence of Aeration on Hydrophobic Organic Contaminant Distribution and Diffusive Flux in Estuarine Sediments. *Environmental Science & Technology* **2003**, *37,* 3547-3554.

Linton, K.B.; Richmond, M.H.; Bevan, R.; Gillespie, W.A. Antibiotic Resistance and R Factors in Coliform Bacilli Isolated from Hospital and Domestic Sewage. *J. Med. Microbiol.* **1974**, *7,* 91-103.

Liu, H.; Zhang, G.; Liu, C.-Q.; Li, L.; Xiang, M. The occurrence of chloramphenicol and tetracyclines in municipal sewage and the Nanming River, Guiyang City, China. *J. Environ. Monit.* **2009**, *11,* 1199-1205.

Liu, L.; Phanikumar, M.S.; Molloy, S.L.; Whitman, R.L.; Shively, D.A.; Nevers, M.B.; Schwab, D.J.; Rose, J.B. Modeling the Transport and Inactivation of E. coli and Enterococci in the Near-Shore Region of Lake Michigan. *Environmental Science & Technology* **2006**, *40,* 5022-5028.

Loferer-Krößbacher, M.; Klima, J.; Psenner, R. Determination of bacterial cell dry mass by transmission electron microscopy and densitometric image analysis. *Applied Environmental Microbiology* **1998**, *64,* 688-694.

Loke, M.-L.; Tjornelund, J.; Halling-Sorensen, B. Determination of the distribution coefficient (logKd) of oxytetracycline, tylosin A, olaquindox and metronidazole in manure, *Chemosphere* **2002**, *48*, 351-361.

Lunestad, B.T.; Goksoeyr, J. Reduction in the antibacterial effect of oxytetracycline in sea water by complex formation with magnesium and calcium. *Diseases of Aquatic Organisms* **1990**, *9*, 67-72.

Mach, P.A.; Grimes, D.J. R-plasmid transfer in a wastewater treatment plant. *Appl. Environ. Microbiol.* **1982**, *44,* 1395-1403.

MacKay, A.A.; Canterbury, B. Oxytetracycline Sorption to Organic Matter by Metal-Bridging. *J. Environ. Qual.* **2005**, *34*, 1964-1971.

McPherson, P.; Gealt, M.A. Isolation of indigenous wastewater bacterial strains capable of mobilizing plasmid pBR325. *Appl. Environ. Microbiol.* **1986**, *51,* 904-909.

Mezrioui, N.; Baleux, B. Resistance patterns of e. coli strains isolated from domestic sewage before and after treatment in both aerobic lagoon and activated sludge. *Water Research* **1994**, *28,* 2399-2406.

Muñoz-Aguayo, J.; Lang, K.S.; LaPara, T.M.; González, G.; Singer, R.S. Evaluating the Effects of Chlortetracycline on the Proliferation of Antibiotic-Resistant Bacteria in a Simulated River Water Ecosystem. *Applied and Environmental Microbiology* **2007**, *73,* 5421-5425.

Murray, G.E.; Tobin, R.S.; Junkins, B.; Kushner, D.J. Effect of chlorination on antibiotic resistance profiles of sewage-related bacteria. *Appl. Environ. Microbiol.* **1984**, *48,* 73–77.

Nygaard, K.; Lunestad, B.T.; Hektoen, H.; Berge, J.A.; Hormazabal, V. Resistance to oxytetracycline, oxolinic acid and furazolidone in bacteria from marine sediments. *Aquaculture* **1992**, *104*, 31-36.

Parveen, S.; Lukasik, J.; Scott, T.M.; Tamplin, M.L.; Portier, K.M.; Sheperd, S.; Braun, K.; Farrah, S.R. Geographical variation in antibiotic resistance profiles of Escherichia coli isolated from swine, poultry, beef and dairy cattle farm water retention ponds in Florida. *Journal of Applied Microbiology* **2006**, *100*, 50-57.

Peak, N.; Knapp, C.W.; Yang, R.K.; Hanfelt, M.M.; Smith, M.S.; Aga, D.S.; Graham, D.W. Abundance of six tetracycline resistance genes in wastewater lagoons at cattle feedlots with different antibiotic use strategies. *Environmental Microbiology* **2007**, *9*, 143-151.

Pei, R.; Kim, S.-C.; Carlson, K.H.; Pruden, A. Effect of River Landscape on the sediment concentrations of antibiotics and corresponding antibiotic resistance genes (ARG). *Water Research* **2006**, *40*, 2427-2435.

Pouliquen, H.; Le Bris, H. Sorption of oxolinic acid and oxytetracycline to marine sediments. *Chemosphere* **1996**, *33*, 801−815.

Pruden, A.; Pei, R.; Storteboom, H.; Carlson, K.H. Antibiotic Resistance Genes as Emerging Contaminants: Studies in Northern Colorado. *Environmental Science & Technology* **2006**, *40,* 7445-7450.

Rabolle, M.; Spliid, N.H. Sorption and mobility of metronidazole, olaquindox, oxytetracycline and tylosin in soil. *Chemosphere* **2000**, *40*, 715−722.

Rose, P.E.; Pedersen, J.A. Fate of oxytetracycline in streams receiving aquaculture discharges: Model simulations. *Environmental Toxicology and Chemistry* **2005**, *24*, 40-50.

Samuelsen, O.B.; Lunestad, B.T.; Ervik, A.; Fjelde, S. Stability of antibacterial agents in an artificial marine aquaculture sediment studied under laboratory conditions. *Aquaculture* **1994**, *126,* 283-290.

Sapkota, A.R.; Curriero, F.C.; Gibson, K.E.; Schwab, K.J. Antibiotic-Resistant Enterococci and Fecal Indicators in Surface Water and Groundwater Impacted by a Concentrated Swine Feeding Operation. *Environ. Health Perspect.* **2007**, *115*, 1040–1045.

Sarmah, A.K.; Meyer, M.T.; Boxall, A.B.A. A global perspective on the use, sales, exposure pathways, occurrence, fate and effects of veterinary antibiotics (VAs) in the environment. *Chemosphere* **2006**, *65*, 725-759.

Sassman, S.A.; Lee, L.S. Sorption of Three Tetracyclines by Several Soils: Assessing the Role of pH and Cation Exchange. *Environmental Science & Technology* **2005**, *39,* 7452-7459.

Sayah, R.S.; Kaneene, J.B.; Johnson, Y.; Miller, R. Patterns of Antimicrobial Resistance Observed in Escherichia coli Isolates Obtained from Domestic- and Wild-Animal Fecal Samples, Human Septage, and Surface Water. *Applied and Environmental Microbiology* **2005**, *71*, 1394-1404.

Schnoor, J.L. *Environmental Modeling: Fate and Transport of Pollutants in Water, Air and Soil*; Wiley: New York, 1996.

Sengelov, G.; Agerso, Y.; Halling-Sorensen, B.; Baloda, S.B.; Andersen, J.S.; Jensen, L.B. Bacterial antibiotic resistance levels in Danish farmLand as a result of treatment with pig manure slurry. *Environment International* **2003**, *28*, 587-595.

Servais, P.; Anzil, A.; Ventresque, C. A simple method for the determination of biodegradable dissolved organic carbon in water. *Applied Environmental Microbiology* **1989**, *55*, 2732-2734.

Sithole, B.B.; Guy, R.D. Models for tetracycline in aquatic environments. *Water, Air, Soil Pollut.* **1987**, *32*, 303−314.

Smith, P.; Samuelsen, O.B. Estimates of the significance of out-washing of oxytetracycline from sediments under Atlantic salmon sea-cages. *Aquaculture* **1996**, *144*, 17-26.

Stewart, F.M.; Levin, B.R. The Population Biology of Bacterial Plasmids: A Priori Conditions for the Existence of Conjugationally Transmitted Factors. *Genetics* **1977**, *87*, 209–228.

Stewart, K.R.; Koditschek, L. Drug-resistance transfer in Escherichia coli in New York Bight sediment. *Marine Pollution Bulletin 1980*, *11*, 130-133.

Storteboom, H.; Arabi, M.; Davis, J.G.; Crimi, B.; Pruden, A. Identification of Antibiotic-Resistance-Gene Molecular Signatures Suitable as Tracers of Pristine River, Urban, and Agricultural Sources. *Environ. Sci. Technol.* **2010**, *44*, 1947–1953.

Tao, R.; Ying, G.-G.; Su, H.-C.; Zhou, H.-W.; Sidhu, J.P.S. Detection of antibiotic resistance and tetracycline resistance genes in Enterobacteriaceae isolated from the Pearl rivers in South China. *Environmental Pollution* **2010**, *158*, 2101-2109.

Thanassi, D.G.; Suh, G.S.; Nikaido, H. Role of outer membrane barrier in efflux-mediated tetracycline resistance of Escherichia coli. *J. Bacteriol.* **1995**, *177*, 998-1007.

Thomann, R.V.; Mueller, J.A. *Principles of Surface Water Quality Modeling and Control*; HarperCollins: New York, NY, 1987.

Thurman, E.M.; Lindsey, M.E. Transport of antibiotics in soil and their potential for groundwater contamination. Presented at 3rd SETAC World Congress, Brighton, UK, May 22−25, 2000.

Tolls, J. Sorption of Veterinary Pharmaceuticals in Soils: A Review. *Environ. Sci. Technol.* **2001**, *35*, 3397–3406.

Turner, P.E. Phenotypic Plasticity in Bacterial Plasmids. *Genetics* **2004**, *167*, 9-20.

Verma, B.; Headley, J.V.; Robarts, R.D. Behaviour and fate of tetracycline in river and wetland waters on the Canadian Northern Great Plains. *J. Environ. Sci. Health. A.* **2007**, *42*, 109-117.

Vital, M.; Hammes, F.; Egli, T. Escherichia coli O157 can grow in natural freshwater at low carbon concentrations. *Environmental Microbiology* **2008**, *10*, 2387-2396.

Warbington, R.C.; Martin, K.L.; Rice, D.A.; Howell, D.G.; Boyd, W.L. A membrane filter method for the enumeration of aquatic ammonifiers. *Water Research* **1987**, *21*, 601-604.

Watkinson, A.J.; Micalizzi, G.B.; Graham, G.M.; Bates, J.B.; Costanzo, S.D.. Antibiotic resistant Escherichia coli in wastewaters, surface waters and oysters from an urban riverine system. *Appl. Environ. Microbiol.* **2007b**, doi:10.1128/AEM.00763-07.

Watkinson, A.J.; Micalizzi, G.R.; Bates, J.R.; Costanzo, S.D. Novel Method for Rapid Assessment of Antibiotic Resistance in Escherichia coli Isolates from Environmental Waters by Use of a Modified Chromogenic Agar. *Applied and Environmental Microbiology* **2007**, *73*, 2224-2229.

Webb, G.F.; D'Agata, E.M.C.; Magal, P.; Ruan, S. *A model of antibiotic-resistant bacterial epidemics in hospitals. Proc. Natl. Acad. Sci. USA* **2005**, *102*, 13343-13348.

Werner, J.J.; Arnold, W.A.; McNeill, K. Water Hardness as a Photochemical Parameter: Tetracycline Photolysis as a Function of Calcium Concentration, Magnesium Concentration, and pH. *Environmental Science & Technology* **2006**, *40*, 7236-7241.

Wetzel, R.G. *Limnology: Lake and River Ecosystems*; 3rd Ed.; Academic Press: San Diego, CA, 2001.

Whitton, B.A. *River ecology*; University of California Press: Berkeley, 1975.

Yang, S.; Carlson, K. Evolution of antibiotic occurrence in a river through pristine, urban and agricultural landscapes. *Water Research* **2003**, *37*, 4645-4656.

Yang, S.; Carlson, K. Routine monitoring of antibiotics in water and wastewater with a radioimmunoassay technique. *Water Research* **2004**, *38*, 3155-3166.

Yang, S.; Cha, J.; Carlson, K. Simultaneous extraction and analysis of 11 tetracycline and sulfonamide antibiotics in influent and effluent domestic wastewater by solid-phase extraction and liquid chromatography-electrospray ionization tandem mass spectrometry. *Journal of Chromatography A* **2005**, *1097*, 40-53.
